# A Metal‐Organic Polyhedron‐to‐Coordination Polymer Transition Revealed by 3D Electron Diffraction

**DOI:** 10.1002/anie.202514527

**Published:** 2025-09-06

**Authors:** Matthew P. Snelgrove, Beatriz Doñagueda Suso, Calum S. Sangster, Khadija Asif, Emma Regincós Martí, David J. Ashworth, Jeremiah P. Tidey, José R. B. Gomes, Miguel Jorge, Alan R. Kennedy, Simon Parsons, Ashleigh J. Fletcher, Gavin A. Craig

**Affiliations:** ^1^ Department of Pure and Applied Chemistry University of Strathclyde Glasgow G1 1RX UK; ^2^ EaStCHEM School of Chemistry and Centre for Science at Extreme Conditions University of Edinburgh Edinburgh EH9 3FJ UK; ^3^ Department of Chemical and Process Engineering University of Strathclyde Glasgow G1 1XJ UK; ^4^ Department of Physics University of Warwick Gibbet Hill Road Coventry CV4 7AL UK; ^5^ Department of Chemistry CICECO‐Aveiro Institute of Materials, University of Aveiro Aveiro 3810‐193 Portugal

**Keywords:** 3D Electron diffraction, Metal‐organic polyhedra, Molecular simulation, Porous materials, Supramolecular chemistry

## Abstract

Porous metal‐organic polyhedra (MOPs) have strong covalent and coordinate bonds that define the intrinsic pore of the cage. The intermolecular interactions between cages tend to be weaker, such that they rearrange during the solvent exchange process preceding gas sorption measurements. The reduction in crystal size that this often causes limits the availability of structural data that could enable understanding of observed gas uptake. Herein, we use 3D electron diffraction (ED) to resolve this problem, and apply this technique to a MOP‐based material that shows cooperative gas capture. 3D ED structure solution reveals both that the MOPs rearrange to form porous 1D polymers, and that these polymers are retained in the activated phase. Molecular simulations using these data suggest gas uptake is facilitated by rotation of functional groups appended to the backbone of the polymers in conjunction with structural expansion as gas is accommodated. Mechanical downsizing of the material leads to the loss of cooperative gas uptake, but a level of porosity is retained, attributed to the conservation of the 1D polymer structure. This work underscores the potential of 3D ED for probing structural transformations in functional supramolecular materials.

## Graphical abstract

Here, we show that 3D electron diffraction (3D ED) is an effective technique to observe structural transformations in porous metal‐organic polyhedra (MOPs), as well as for obtaining the structure of the activated phases of these materials. A MOP‐to‐coordination polymer transition is revealed, and explains the reduction in cooperative gas uptake by the material as it is mechanically downsized via ball milling.

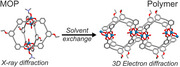

## Introduction

**Figure 1:**
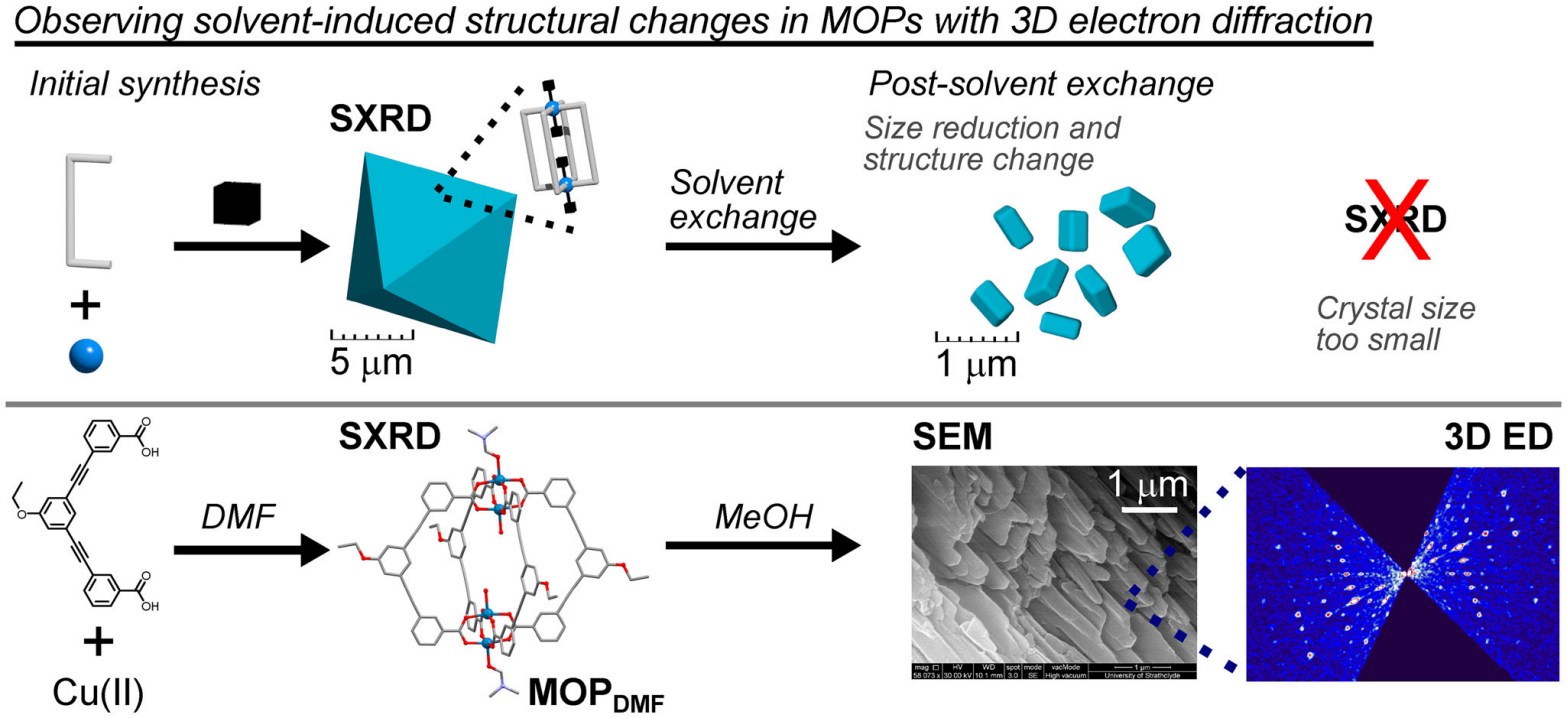
(Top) The initial synthesis of MOPs in non‐volatile solvents aims to obtain large single crystals, where single crystal X‐ray diffraction (SXRD) reveals the molecular structure, but solvent exchange processes prior to gas sorption reduce this crystal size to sub‐micron scale, rendering MOPs unsuitable for SXRD. (Bottom) Here, we use 3D ED to observe these structural changes in the MOP **MOP_DMF_**, where solvent exchange with MeOH reduces the material to aggregates of thin, high aspect ratio crystallites, as shown by SEM images.

Porous molecular solids are widely studied supramolecular materials. Porous organic cages (POCs) and metal‐organic polyhedra or metal‐organic cages (MOPs or MOCs) present intrinsic pores associated with the molecular cage unit, which is synthesised using bottom‐up strategies to target specific cage geometries.^[^ ^1^, ^2^, ^3^, ^4^, ^5^, ^6^ ^]^ These materials may, therefore, present extrinsic porosity that originates in molecular packing, in addition to the intrinsic porosity arising from the cage geometry. To conserve the intrinsic pore in the activated phase of MOPs, synthetic approaches to their formation generally use rigid, functionalised aromatic ligands that show highest solubility in polar, high boiling point solvents such as dimethylformamide (DMF), dimethylacetamide (DMA), and dimethylsulfoxide (DMSO).^[^ ^7^, ^8^, ^9^, ^10^, ^11^, ^12^, ^13^, ^14^, ^15^, ^16^, ^17^, ^18^ ^]^ The as‐synthesised MOP geometry is then confirmed using single crystal X‐ray diffraction (Figure 1). To facilitate activation of the material and measurement of its porosity, the solvent used in the synthesis is exchanged for a more volatile solvent, such as methanol or tetrahydrofuran – a strategy that was outlined for the early examples of successful measurement of surface areas in MOPs.^[^ ^19^, ^20^ ^]^ This widely‐adopted method has enabled the gas uptake of a wide range of MOPs to be measured.^[^ ^21^ ^]^ However, the generally weak intermolecular interactions between cages renders them prone to solvent‐driven changes in crystal packing.^[^ ^22^, ^23^ ^]^ At the same time, the particle size of the materials and/or crystallinity can be reduced to such an extent that they are unsuitable for single crystal X‐ray diffraction experiments. The retention of the cage geometry is then inferred from methods such as infra‐red (IR) spectroscopy or observation of microporosity in gas sorption isotherms, rather than direct visualisation.

3D electron diffraction (3D ED, or microcrystal electron diffraction, microED) has become a valuable tool in the field of porous materials to obtain structures where small crystal size inhibits the use of single crystal X‐ray diffraction, even at synchrotron sources.^[^ ^24^, ^25^, ^26^, ^27^ ^]^ It has been used, whether on its own or in combination with powder X‐ray diffraction, to solve the structures of zeolites,^[^ ^28^, ^29^ ^]^ covalent‐organic frameworks,^[^ ^30^ ^]^ and metal‐organic frameworks.^[^ ^31^, ^32^, ^33^ ^]^ An exciting application of 3D ED is for cases where it can shed light on dynamic systems. For example, it has been used to probe linker rotation in the frameworks MIL‐140C and UiO‐67^[^ ^34^ ^]^; understand the adsorption steps in atropisomers of COF‐320^[^ ^35^ ^]^; and prove topochemical polymerisation in MOFs.^[^ ^36^ ^]^ In a rare example studying porous molecular materials, Cui *et al*. observed reorientation of anthracene units within a 2D hydrogen‐bonded organic framework upon activation.^[^ ^37^ ^]^ To the best of our knowledge, there are no reports that use 3D ED to study intrinsically porous molecules such as MOPs.

Herein, we show that 3D ED is an effective method to obtain the structures of MOP‐based materials. Previous work on the cage [Cu_4_(EtOL)_4_(DMF)_2_(H_2_O)_2_]·n(DMF), **MOP_DMF_**, where EtOL^2−^ is 3,3′‐((5‐ethoxy‐1,3‐phenylene)*bis*(ethyne‐2,1‐diyl))dibenzoate, found that the activated material showed cooperative gas uptake for CO_2_, but the structures of the materials were unknown beyond the as‐synthesised single crystals (MOP structure shown in Figure 1).^[^ ^38^ ^]^ Here, we use 3D ED to not only obtain the structure of the solvent‐exchanged phase, but also to reveal the structure of the activated phase by in situ activation of this phase. These experiments show that the as‐synthesised MOP converts to a one‐dimensional coordination polymer upon solvent exchange, and this structure is retained in the activated phase. Theoretical simulations made possible by the 3D ED structure solution suggest that the initial gas uptake is driven by a combination of structural expansion and rotation of the ethoxy‐ groups on the polymers. Loss of crystallinity induced by ball milling is then shown to affect the cooperative step in the gas sorption isotherms, while the polymeric structure is retained. The insight provided by 3D ED allows this to be attributed to a loss of ordered packing of the coordination polymer in this particular case.

## Results and Discussion

For materials that show cooperative behaviour in the solid state, such as spin switching, it is well known that the crystal size of the material affects the nature of the transition between the possible phases.^[^ ^39^, ^40^, ^41^ ^]^ This phenomenon has also been described for MOFs.^[^ ^42^, ^43^, ^44^, ^45^, ^46^ ^]^ Given the cooperative step that had been observed in the CO_2_ gas sorption isotherms of the activated phase obtained from **MOP_DMF_**, we set out to assess the effect of crystallinity and crystal size on this step. Initially, we sought to control the crystal size of the materials through a range of synthetic methods (e.g., changes in concentration, use of modulators, temperature of crystallisation) with little success. Therefore, to reduce the crystal size, we used ball milling of the MeOH‐exchanged phase of **MOP_DMF_**. The synthesis of **MOP_DMF_** followed the reported procedure, which involves reaction of the ligand 3,3′‐((5‐ethoxy‐1,3‐phenylene)bis(ethyne‐2,1‐diyl))dibenzoic acid with copper(II) acetate in dimethylformamide.^[^ ^38^ ^]^ Ahead of gas sorption experiments, **MOP_DMF_** is soaked in MeOH, as this more volatile solvent facilitates activation of the material. However, this process induces a severe reduction in the crystallite size, yielding a distinct phase in the form of a highly crystalline powder. The morphology of the crystallites obtained from this solvent exchange process was not previously reported. Inspection of the crystallites of the material that had been soaked in MeOH using scanning electron microscopy (SEM) show that the solvent‐induced transformation leads to thin, finger‐like crystallites with high aspect ratios: although one dimension of the particles reaches micron scale, the thickness of the crystallites is around 100–300 nm (Figure 1). The morphology of the crystallites lends them a tendency to aggregate into stacks. Despite the aggregation observed in the SEM images, the morphology of the individual crystallites is favourable for 3D electron diffraction studies.

**Figure 2:**
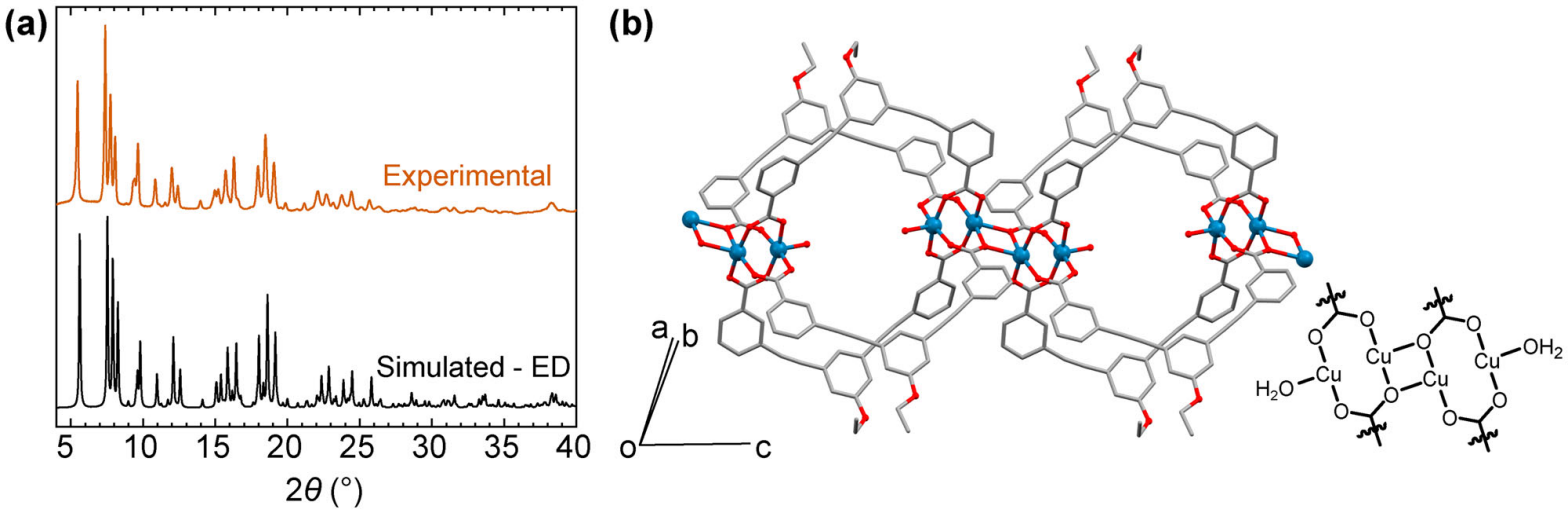
a) Comparison of the powder X‐ray diffractogram simulated from the 3D ED structure and the experimentally obtained data for ***p*MOP_H2O_**. b) View of one of the polymer chains that form along the *c*‐axis in ***p*MOP_H2O_** through the mutual coordination of paddlewheel units on neighbouring MOPs, with ChemDraw representation to clarify the coordination mode. Hydrogen atoms are omitted for clarity.

We were able to isolate suitable samples for 3D ED by lightly grinding polycrystalline powders between two glass slides and dispersing them dry onto a copper‐supported holey carbon transmission electron microscopy (TEM) grid. A range of crystallites were probed for this, and the data presented here were obtained on crystallites that were isolated from a sample that was ground at 12 Hz using a ball mill. Data were collected at 100 K to obtain the structure of this phase (see Supporting Information for full details of all 3D ED experiments, and exemplar images of the crystallites and diffraction). As shown in Figure 2, the solvent exchange process induces a structural transition from the discrete lantern‐type cage – as seen in Figure 1 – to a one‐dimensional coordination polymer composed of MOPs, with a formula of [Cu_4_(EtOL)_4_(H_2_O)_2_]*_n_*, which we label ***p*MOP_H2O_**. The material crystallises in the triclinic space group $P\bar{1}$, where the asymmetric unit contains one half of a monomer unit, and the experimental powder X‐ray diffraction data for ***p*MOP_H2O_** show excellent agreement with the simulated pattern based on the 3D ED structure solution (Figure 2a; view of asymmetric unit and Pawley refinement are shown in Figures S2 and S3). The lantern‐type molecular unit of the as‐synthesised phases is retained but mutual coordination of Cu(II)‐paddlewheel moieties on neighbouring cages connects the MOPs into polymers that extend along the *c*‐axis of the unit cell. The bridge between MOP units consists of a Cu_2_O_2_ ring. This structural motif has been described for other paddlewheel‐based compounds,^[^ ^47^, ^48^, ^49^, ^50^ ^]^ and was reported in the 3D dehydrated phase of the MOF STAM‐17‐OEt, where water molecules could re‐insert into the axial site of the paddlewheel and drive a transition back to the 2D hydrated Kagome framework.^[^ ^51^ ^]^ It has been described in one case to link cuboctahedral MOPs where hydrophobic solvents (CH_2_Cl_2_, benzene) favoured the discrete MOP and hydrophilic solvents (DMF) favoured the formation of polymers^[^ ^52^ ^]^ but, generally, the mutual coordination of identical MOPs to form a polymer is unusual.^[^ ^53^, ^54^, ^55^ ^]^ We found that these polymers could be readily redissolved in DMA and crystallise within a few hours, returning to a discrete molecular lantern complex, **MOP_DMA_** (data presented in SI). The only remaining coordination site on the paddlewheels in ***p*MOP_H2O_** is the axial site on the inner Cu(II) ion that points into the pore of the cage, and this is occupied by a water molecule. The water molecule induces a very tightly packed structure by forming hydrogen bonding interactions with the O‐atoms of the pendant ethoxy‐ groups on neighbouring polymers [O1_H2O_···O1B_ethoxy_ = 2.788(11) Å; Figure S7]. In this way, neighbouring polymers interdigitate, and there are no obvious porous channels within the structure.

**Figure 3:**
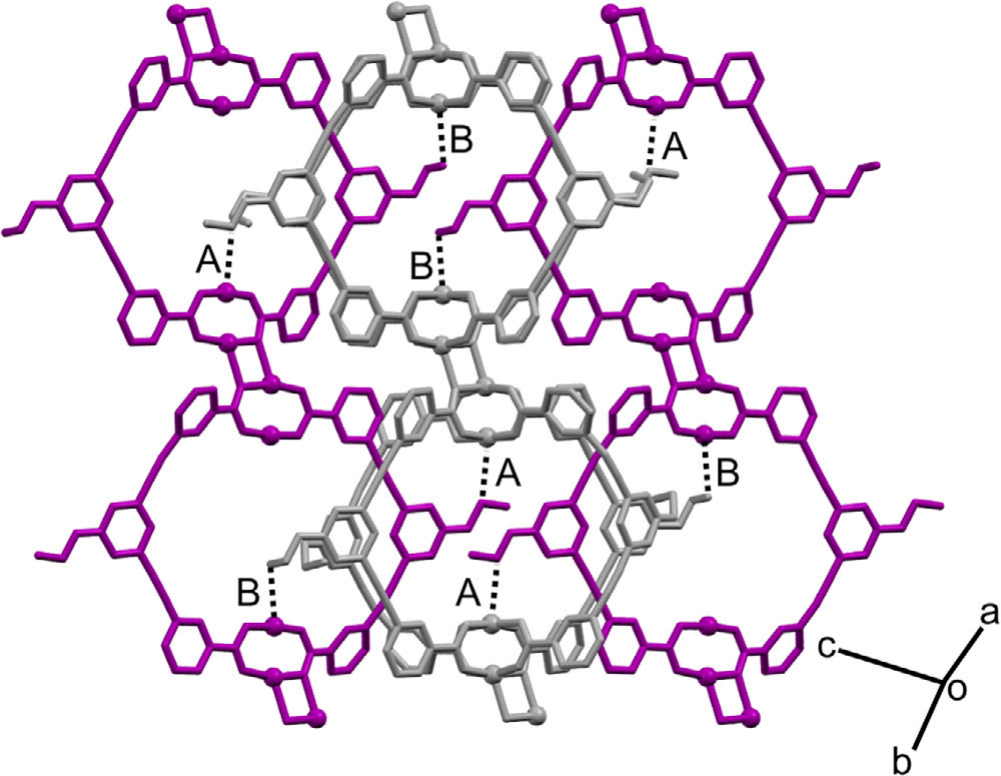
View of three adjacent polymers in ***p*MOP**, where polymers have been coloured alternately for clarity and hydrogen atoms are omitted. Dashed lines correspond to Cu···C distances mentioned in the text.

Next, we were able to activate ***p*MOP_H2O_**, in a rare example of an activated phase observed using 3D ED.^[^ ^56^ ^]^ The crystallites were heated to 350 K in the airlock of the diffractometer, before being cooled to room temperature, where data collected showed a new unit cell. The crystallites were left under vacuum (∼5 × 10^−6^ mbar) overnight in a vacuum station separate to the 3D ED instrument, before being reintroduced to the diffractometer and cooled to 150 K for full data collection (an exemplar image of the crystallites and the diffraction is given in Figure S8). The structure of this activated phase, ***p*MOP**, was obtained by merging five data‐sets (SI Table S3). The data show that ***p*MOP** also crystallises in the triclinic space group $P\bar{1}$. Again, excellent agreement is found between the simulated PXRD pattern and that obtained experimentally (Figures S9 and S10). As can be seen in Figure 3, the polymeric structure observed in ***p*MOP_H2O_** is retained in ***p*MOP**, although the composition of the polymer is instead [Cu_4_(EtOL)_4_]*_n_*, following loss of the coordinated water molecules during activation. In this structure, the asymmetric unit consists of two halves of neighbouring pores in the polymer (see Figure S11). The interdigitated structure that leads to an apparently non‐porous phase in ***p*MOP_H2O_** is also seen in ***p*MOP**. The removal of the terminal water molecule from the inner site leads to an open metal site at the Cu(II) ion, but this space is crowded by the ethoxy‐ groups of neighbouring polymer chains [Cu4···C2B = 3.73(1) Å or Cu2···C1C = 3.28(2) Å, shown in Figure 3 as A and B, respectively].

**Figure 4:**
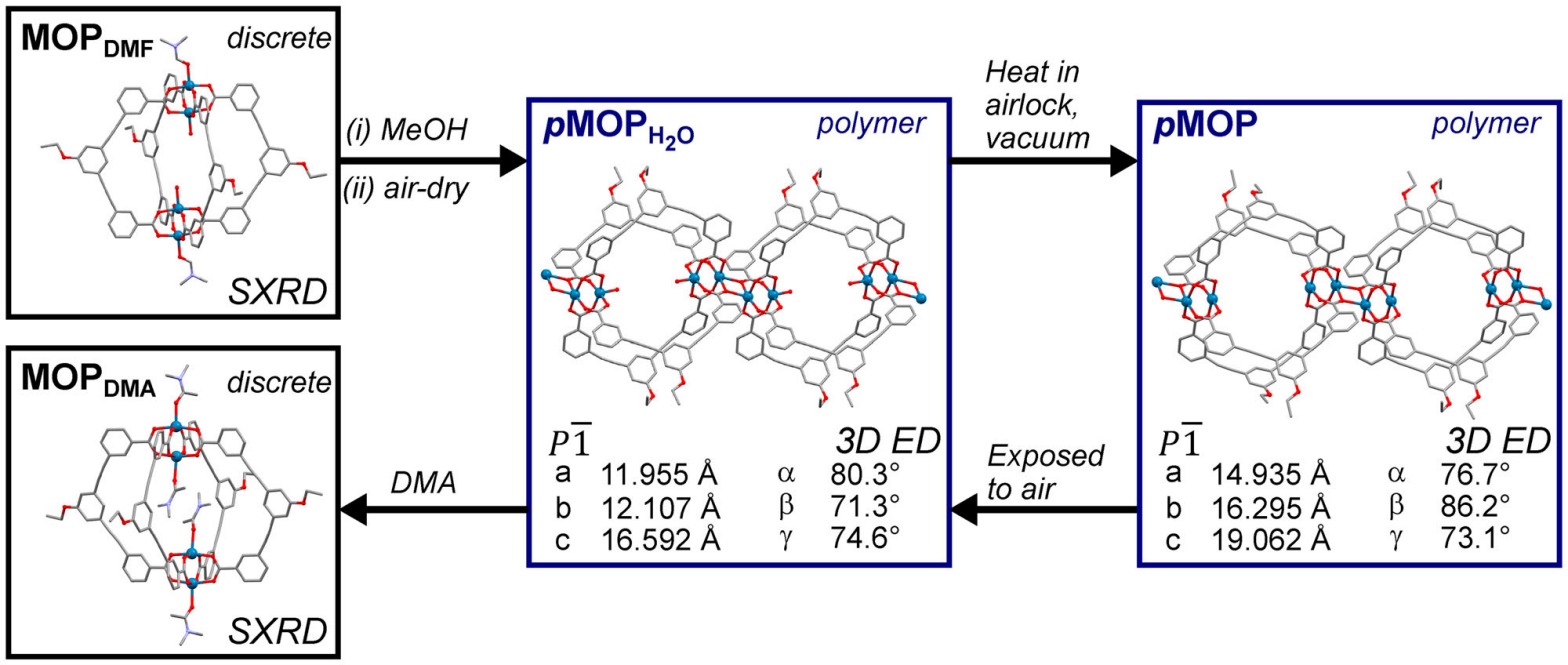
Summary of the structural transformations reported here, indicating how they are induced, and the crystallographic technique used to obtain the structure.

This series of structural transformations is summarised in Figure 4. The discrete cage molecule, **MOP_DMF_**, is transformed into a one‐dimensional polymer, ***p*MOP_H2O_**, during the solvent exchange process that is used prior to gas sorption measurements, as revealed by 3D ED. This polymer can be broken back into discrete MOPs using DMA, to give the phase **MOP_DMA_**, where large single crystals allow the use of SXRD to determine the structure. The hydrated polymer ***p*MOP_H2O_** could then be activated by heating in the airlock of the electron diffractometer to yield the structure of ***p*MOP**.

**Figure 5:**
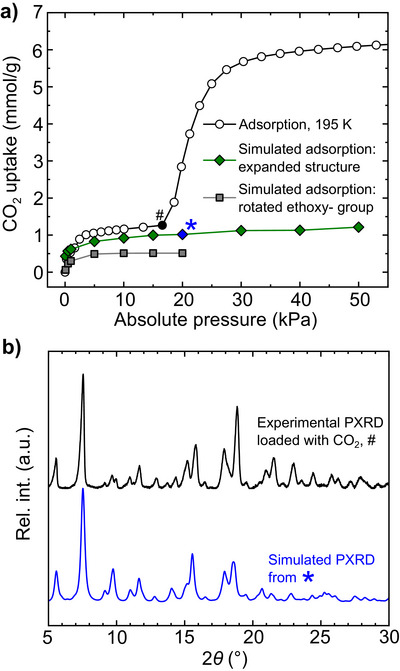
a) Comparison of the experimental CO_2_ isotherm measured at 195 K with the simulated isotherm calculated for ***p*MOP** when expanded by 4%, and the simulated isotherm obtained upon rotation of the ethoxy‐ group. b) The experimental powder X‐ray diffractogram for ***p*MOP** loaded at *ca*. 17 kPa of CO_2_ and the simulated diffractogram obtained from the calculated structure at 20 kPa. The experimental data shown in a,b) were reported in reference 38, and are used here under a Creative Commons Attribution 3.0 Unported License.

Using the structural model for ***p*MOP** obtained from 3D ED as a starting point, we performed geometric porosity characterisation and Grand Canonical Monte Carlo (GCMC) simulations of adsorption, using PoreBlazer^[^ ^57^ ^]^ and RASPA,^[^ ^58^ ^]^ respectively, in order to understand the gas uptake that had been reported for this material (see Supporting Information for detail on procedure). Initial GCMC simulations of the CO_2_ isotherm at 195 K across a pressure range of 0–60 kPa that used the experimental ***p*MOP** structure showed negligible gas uptake (Figure S12). This was rationalised on the basis that ***p*MOP** shows very tight packing of the individual chains, with no immediately obvious accessible porosity. We therefore posited two possible sources of movement within the material that would generate porosity and that were not reflected in this initial simulation. First, rotation of the ethoxy‐ groups bonded to the edges of the MOP units that lie within the pores of adjacent polymers was considered. To test this, we used the structure observed in 3D ED and created new configurations by rotating each ethoxy‐ group in increments of approximately 90° (see the SI for details of these models), before optimising each geometry using density functional theory (DFT) without symmetry restraints and using these structures to simulate the isotherms. Figure 5 presents the results for one of these models, showing uptake in the low‐pressure region similar to the experimental isotherm at 195 K reported previously (the simulated isotherms from the other models generated by this approach are shown in Figure S12). The uptake is no longer negligible, as in the simulation for the experimental structure, but is still markedly lower than that observed experimentally, suggesting that rotation alone cannot explain the adsorption mechanism. Second, we tested the relative movement of the polymers themselves such that they retain their structure but expand the space between them. To test the effect of expansion, the unit cell of ***p*MOP** was expanded uniformly along the unit cell vectors by 2%, 4%, 6%, and 8%; the atomic positions were optimised; and the resulting structures used to simulate the uptake of CO_2_.

Above 6%, the calculated bond order and the length of the Cu‐O bond that links adjacent paddlewheels into the 1D polymer markedly decreases and increases, respectively, suggesting rupture of the chains (Figure S13). Therefore, the structure that had been expanded by 4% was used for simulation of the isotherm (see Figure S14 for the simulations using the other expanded structures). The result shows nearly quantitative agreement with the first step that is observed in the experimental data (Figure 5a). We attribute the slight underestimation in the simulation to the simplified level of theory used, which does not account for specific coordination‐type interactions between CO_2_ and the open coordination site on Cu (see the SI for detailed discussion of this point).^[^ ^59^ ^]^ Using the final configuration obtained from GCMC, it is possible to simulate the corresponding powder X‐ray diffractogram (Figure 5b). The simulated diffractogram for 20 kPa is similar to the experimental powder X‐ray diffraction data for a sample that was loaded with CO_2_ at *ca*. 17 kPa, and the Pawley refinement of these experimental data support the expansion of the unit cell as CO_2_ is captured (Figure S15). Our analysis thus suggests that the 1D polymeric structure observed in ***p*MOP** is retained as the material adsorbs CO_2_ in the first step of the isotherm, and that this uptake is facilitated by a combination of rotation of the ethoxy‐ groups on the polymers and expansion of the structure as CO_2_ is accommodated.

**Figure 6:**
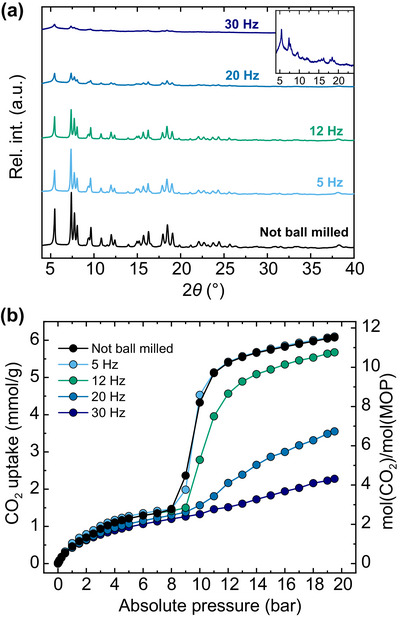
a) PXRD data for ***p*MOP_H2O_** subsequent to ball milling at the frequencies indicated. The inset shows a magnified view of the region between 4 and 24° in 2*θ* for the sample that had been ball milled at 30 Hz. b) CO_2_ adsorption isotherms collected for these samples at 273 K, over the pressure range 0–20 bar. For clarity, only the adsorption branches are shown, with the desorption presented in the Supporting Information.

To reduce the crystallinity of ***p*MOP_H2O_** and determine the effect on the gas uptake, samples were first ball milled at four frequencies (5, 12, 20, and 30 Hz) for 5 min. The resulting powder X‐ray diffraction data for these samples are shown in Figure 6 (post‐gas sorption data and IR spectra for these are provided in Figures S16–S21). A significant loss in crystallinity of the materials is not observed until milling above 12 Hz. The reduction in crystallinity and particle size is reflected in a broadening of the diffraction peaks, and is observed in the corresponding SEM images for the materials (Figures S22–S30). Below 12 Hz, the most intense peak in PXRD is that found at 2*θ* = 7.4°, which can be identified as corresponding to the [0 1 0] direction of ***p*MOP_H2O_**, in which the polymers interdigitate. Above 12 Hz, the most intense peak in the diffractograms is that found at 2*θ* = 5.5°, which corresponds to the [0 0 1] direction, along which the polymers extend. These structural assignments are based on the 3D ED structural models, and highlight the value of the technique for proving the retention of structure in mechanically downsized materials.^[^ ^60^ ^]^ The uptake of CO_2_ for these materials was then measured at 273 K over the pressure range 0–20 bar. All of the samples show comparable behaviour in the pressure range 0 to *ca*. 8 bar: a gradual increase in uptake, the magnitude of which at 8 bar decreases slightly with increased ball milling. The non‐ball milled sample displays an abrupt step in the adsorption isotherm over the range 8–11 bar, with an increase from 1.5 to 5.2 mmol g^−1^, before increasing more slowly with pressure and attaining a maximum uptake of 6.1 mmol g^−1^. The desorption branch traces out a hysteresis loop, with the downward step taking place between 8 and 5 bar (5 mmol g^−1^ decreasing to 1.3 mmol g^−1^, respectively; data presented in Figure S31). This behaviour was replicated in the sample that was ball milled at 5 Hz. Ball milling at higher frequencies led to a significant flattening of the step in the isotherm and a shift towards slightly higher pressures for its onset. The sample that had been ball milled at 12 Hz presented a step at around 9 bar, and attained a maximum uptake of 5.7 mmol g^−1^, while the maximum uptakes observed after ball milling at 20 and 30 Hz were 3.6 and 2.3 mmol g^−1^, respectively. Comparable flattening of the hysteresis loops formed by the desorption branches was also observed (Figures S33–S35). Even at higher frequencies of ball milling, a peak in the powder X‐ray diffraction data is observable at 2*θ* = 5.5°. Based on the results of the theoretical calculations and data from 3D ED, it is proposed that the 1D polymer structures found in ***p*MOP** are retained after ball milling, but the loss of the highly crystalline packing between polymers inhibits the cooperative step that is observed in the sample that had not been ground.

## Conclusion

In summary, here we have used 3D ED structure solution to reveal structural transformations in a supramolecular cage. In this case, 3D ED has shown the rearrangement of a MOP into 1D polymers, and an understanding of at least the initial CO_2_ uptake by the system could be obtained from computational studies based on the 3D ED structural models. Central to the computational studies was the report here of the activated phase of the material. The loss of cooperative gas uptake upon grinding of the material could then be rationalised as due to the retention of the 1D polymer structure at the same time as the ordered packing of these chains was reduced. For this particular system, future work could look at probing the precise mechanism of the MOP‐to‐polymer transition, which would require the rearrangement to be tracked as the concentration of MeOH was increased in the solvent exchange process; and the development of computational models that capture the step in gas sorption at high pressures. For the particular field of porous MOPs, particle size and morphology subsequent to solvent exchange are under‐reported, meaning that there are potentially many materials for which 3D ED could shed light on structural transformations; inform computational studies; and enable structure‐function relationships to be developed. More generally, this work underscores the potential for 3D ED to probe structural transformations in functional supramolecular materials.

## Supporting Information

The authors have cited additional references within the Supporting Information.^[^ ^57^, ^58^, ^61^, ^62^, ^63^, ^64^, ^65^, ^66^, ^67^, ^68^, ^69^, ^70^, ^71^, ^72^, ^73^, ^74^, ^75^, ^76^, ^77^, ^78^, ^79^, ^80^ ^]^ The structural data have been deposited with the Cambridge Crystallographic Data Centre.^81^

## Conflict of Interests

The authors declare no conflict of interest.

## Supporting information



Supporting information

Supporting information

## Data Availability

The raw data that support the findings of this study are openly available from the University of Strathclyde KnowledgeBase at https://doi.org/10.15129/2845ffc9‐8026‐41a8‐ae38‐c2e38fbc01ff.
