# Supplementary material for: A Metal‐Organic Polyhedron‐to‐Coordination Polymer Transition Revealed by 3D Electron Diffraction
Source: Angew Chem Int Ed Engl. 2025 Sep 6;64(43):e202514527. doi: 10.1002/anie.202514527 (PMC12535363; doi:10.1002/anie.202514527)
Supplement: Supplementary file 1 — Supporting information [file ANIE-64-e202514527-s002.pdf]

A metal-organic polyhedron-to-coordination polymer transition revealed by 3D electron diffraction

Matthew P. Snelgrove,[1] Beatriz Doñagueda Suso,[1] Calum S. Sangster,[2] Khadija Asif,[3] Emma Regincós Martí,[1] David J. Ashworth,[3] Jeremiah P. Tidey,[4] José R. B. Gomes,[5] Miguel Jorge,[3] Alan R. Kennedy,[1] Simon Parsons,[2] Ashleigh J. Fletcher,[3] and Gavin A. Craig[1]*

[1]Department of Pure and Applied Chemistry, University of Strathclyde, Glasgow, United Kingdom, G1 1RX. gavin.craig@strath.ac.uk

[2]EaStCHEM, School of Chemistry and Centre for Science at Extreme Conditions, University of Edinburgh, Edinburgh, United Kingdom, EH9 3FJ.

[3]Department of Chemical and Process Engineering, University of Strathclyde, Glasgow, United Kingdom, G1 1XJ.

[4]Department of Physics, University of Warwick, Gibbet Hill Road, Coventry, United Kingdom, CV4 7AL.

[5]Department of Chemistry, CICECO-Aveiro Institute of Materials, University of Aveiro, 3810-193 Aveiro, Portugal

Experimental details

Synthesis

The samples **MOP$_{DMF}$** were synthesised following the procedure reported in reference 38 of the paper.

For the ball milled samples described in the main manuscript, subsequent to solvent exchange with MeOH, samples of ***p*MOP$_{H2O}$** were loaded into a 2 mL Eppendorf with one 5 mm diameter stainless steel ball bearing. The tube was loaded on to a Retsch Mixer Mill MM400 at room temperature, and then milled for 5 minutes at the frequencies indicated in the manuscript.

The phase **MOP$_{DMA}$** was obtained by adding ***p*MOP$_{H2O}$** (50 mg) to DMA (1 mL) and gently heating until full dissolution, to give a deeply coloured green-blue solution. Large crystals formed overnight, collected *via* vacuum filtration and washed thoroughly with DMA, to give the named compound (32 mg).

Physical characterisation

Fourier-Transformed infra-red spectra were collected using a NICOLET iS5 Thermo Scientific spectrometer, with parameters set as 48 scans and a resolution of 4 cm^{-1}. For SEM images, samples were coated with gold using a Leica EM ACE 200 Sputter Coater. SEM images were taken with a JEOL JSM-IT100 with a tungsten source and operating at 20 kV and 40 mA current. All powder X-ray diffraction data (PXRD) were collected in the Centre for Continuous Manufacturing and Advanced Crystallisation (CMAC), at the University of Strathclyde. Powder X-Ray Diffraction (PXRD) data were collected at room temperature over a flat plate using a poly(methyl methacrylate) holder. The instrument used was a Bruker D8 Discover in a $\vartheta/2\vartheta$ Bragg-Brentano reflection geometry using a Cu K_α source (λ = 1.54056 Å). The PXRD data presented in Figures 3 and 4 of the main manuscript correspond to measurements run in capillaries at room temperature on a Bruker D8 Advance equipped with a Cu K_α source (λ = 1.54056 Å). For the activated sample ***p*MOP**, a powder sample of ***p*MOP$_{H2O}$** was heated under vacuum to 120°C on a Schlenk line, loaded into a capillary and then flame-sealed, prior to mounting on the diffractometer. CO_2 sorption isotherms were measured at 273 K using an IGA-003 gravimetric gas sorption analyser from Hiden Isochema Ltd. Samples were activated *in situ* under vacuum at 120°C for 10 hours prior to sorption analysis.

Single crystal X-ray diffraction

Single crystal X-ray diffraction measurements on ***p*MOP$_{H2O}$** were made with monochromatic Cu radiation (λ = 1.54184 Å) using a Rigaku Synergy-i diffractometer. Raw data processing utilised the program CrysalisPro.[1] The structures were refined against F^2 to convergence using all unique reflections (in hklf 5 format) and the program Shelxl,[2] as implemented within WinGX.[3] The structure was treated as a two component twin by a rotation of 180 ° about direction 001. The twin scale factor refined to 0.2833(26). Much of the included

solvent was disordered and/or partially present. Occupancy factors for solvent molecules were set based upon the results of trial calculations. Both disordered solvent molecules and disordered parts of the ligand were treated with appropriate restraints and constraints to ensure that each approximated to normal geometry and displacement behaviour. It is likely that the current model underestimates the total solvent load. Selected crystallographic and refinement parameters are given in Table S1 and full information in .cif format has been deposited with the CCDC as reference number 2469376.

Table S1. Selected Crystallographic and Refinement Parameters for **MOP$_{DMA}$**.

**Compound**	**MOP$_{DMA}$**
**CCDC ref. no.**	2469376
**Formula**	$C_{142.33}H_{150.25}Cu_4N_{9.58}O_{29.58}$
**Form. Wt.**	2722.62
**Crystal system**	Triclinic
**Space group**	*P*-1
**Temp. (K)**	150(2)
***a* (Å)**	17.6359(4)
***b* (Å)**	18.0833(5)
***c* (Å)**	27.0228(3)
***α* (°)**	88.497(2)
***β* (°)**	89.830(1)
***γ* (°)**	63.521(2)
**Volume (Å^3)**	7710.9(3)
Z	2
**Reflections**	103232
**Observed Reflections**	73152
**Completeness (%)**	99.8
**2Θmax (°)**	140.00
**No. Parameters**	1748
**No. Restraints**	329
S	1.405
***R* [on *F*, obs refs only]**	0.1257
***ωR* [on *F*^{2}, all data]**	0.3808
**Residuals (eÅ^{-3})**	1.618/-1.194

3D electron diffraction

For ***p*MOP$_{H2O}$**, sample was lightly ground between two glass slides and dispersed dry onto a copper-supported holey carbon TEM grid; loaded into the Rigaku XtaLAB Synergy-ED electron diffractometer sample airlock at 215 K to sublime ice; and then cooled to 185 K; and then inserted into the column and cooled to 100 K for data collection. To obtain the data for the activated structure ***p*MOP**, these samples were heated to 350 K in the airlock of the diffractometer, before being cooled to room temperature, where data collected showed a

new unit cell. The crystallites were left under vacuum (~5×10^{-6} mbar) overnight in a vacuum station separate to the 3D ED instrument, before being reintroduced to the diffractometer at room temperature, and then cooled to 150 K for full data collection.

All sample loading used a Gatan Elsa model 698 cryo holder. The instrument was operated at 200 kV and equipped with a Rigaku HyPix-ED hybrid pixel array area detector. In each case, data for a range of crystallites were surveyed using continuous rotation electron diffraction with a selected area of ~2 µm diameter at the image plane using CrysAlisPRO (version 1.171.44.70a).[1] The reported data are, in both cases, subsequently taken from the merging of five datasets of comparable quality over tilts of between 94 and 127.5° from crystallites appearing as blocks with dimensions in the order of 0.3 – 1 µm and using frame scan widths of 0.25°. Datasets were in each case individually indexed and integrated, and subsequently merged and scaled together for each model using CrysAlisPRO (version 1.171.44.115a for ***p*MOP$_{H2O}$**; and version 1.171.44.91a for ***p*MOP**)[1] and SCALE3 ABSPACK, implemented therein.

The structures were solved using ShelXT[2] and refined in the kinematic approximation using Olex2.refine[4] as implemented in Olex2 (version 1.5-ac7-016, compiled 2025.05.29 svn.r8cd99b3d for Rigaku Oxford Diffraction, GUI svn.r7254)[5] using published scattering factors.[6] An extinction correction was applied in each case to broadly account for the impact of multiple diffraction with further omission of particularly outlying reflections in the final stages of the refinement, treated as either significantly affected by multiple scattering, or being either in some way obscured during collection or by limitations in the automated outlier rejections otherwise used. All chemically equivalent covalent bonds in each model are restrained to have similar distances. In all cases, C-H hydrogen atoms were placed at geometrically constrained positions at neutron distances. Water protons in ***p*MOP$_{H2O}$** were initially placed geometrically and their position refined before the removal of geometric constraints and free refinement in the presence of 1,2 distance similarity restraints. All non-H atoms are treated anisotropically with rigid bond restraints applied to better model those displacement parameters; hydrogen atoms were treated with riding isotropic displacement parameters. Complete experimental and refinement information are contained in the deposited CIFs along with structure factors and embedded .RES files. These are deposited in the CSD with CCDC reference codes CCDC 2457254 and 2457255. Tables S2 and S3 report experimental parameters from the merged refinement and individual datasets for ***p*MOP$_{H2O}$** and ***p*MOP**, respectively.

Table S2. Experimental data table for ***p*MOP**$_{H2O}$.

Crystal data	
Chemical formula	$C_{104}H_{68}Cu_4O_{22}$
M_r	1923.85
Crystal system, space group	Triclinic, *P*-1
Temperature (K)	100(5)
a, *b*, *c* (Å)	11.9545(9), 12.1069(6), 16.5916(7)
α, β, γ (°)	80.316(4), 71.254(5), 74.625(5)
V (Å^3)	2183.5(2)
Z	1
Radiation type, wavelength	Electron, λ = 0.02508 Å
**Data collection**	
Scan range (°)	See _diffrn_measurement_details in CIFs for individual component angular ranges.
No. reflections (meas., indep., obs [I ≥ 2u(I)]), completeness	44915, 8330, 6182, 94.4%
R_{int}	0.201
Resolution in $(\sin \theta/\lambda)_{max}$ (Å^{-1}), *d*-spacing (Å)	0.626, 0.80
**Refinement**	
R_1, wR_2 ($[F^2 > 2\sigma(F^2)]$)	0.1687, 0.3264
R_1, wR_2 (all)	0.2092, 0.3478
GoF(*S*) (inc., excl. restraints)	0.9997, 1.0373
No. reflections, rejections, completeness	8267, 63, 93.6%
No. parameters, restraints	595, 588
$\Delta\varphi_{max}$, $\Delta\varphi_{min}$ (as output by Olex2.refine, 1.5-ac07-016)	1.24, −1.03

Table S3. Experimental data table for ***p*MOP**.

Crystal data	
Chemical formula	$C_{104}H_{64}Cu_4O_{20}$
M_r	1886.81
Crystal system, space group	Triclinic, $P-1$
Temperature (K)	150
a, *b*, *c* (Å)	14.9348(7), 16.2954(9), 19.0622(8)
α, β, γ (°)	76.688(4), 86.248(4), 73.103(4)
V (Å^3)	4319.6(4)
Z	2
Radiation type, wavelength	Electron, λ = 0.02508 Å
**Data collection**	
Scan range (°)	See _diffrn_measurement_details in CIFs for individual component angular ranges.
No. reflections (meas., indep., obs [I ≥ 2u(I)]), completeness	90500, 14696, 8656, 95.5%
R_{int}	0.236
Resolution in (sin θ/λ)$_{max}$ (Å^{-1}), *d*-spacing (Å)	0.598, 0.84
**Refinement**	
R_1, wR_2, [$F^2 > 2\sigma(F^2)$]	0.1757, 0.3933
R_1, wR_2, [all]	0.2342, 0.4260
GoF(S) (inc., exlc. restraints)	1.0216, 1.0657
No. reflections, rejections, completeness	14617, 79, 95.0%
No. parameters, restraints	1157, 1184
$\Delta\varphi_{max}$, $\Delta\varphi_{min}$ (as output by Olex2.refine, 1.5-ac07-13)	1.04, −1.08

Simulation details

Density Functional Theory calculations

Initially, the structure for the activated ***p*MOP** was optimised by Density Functional Theory (DFT), starting from the structure resolved experimentally by 3D ED. This was done keeping the unit cell parameters fixed and optimizing only the atomic positions. Density functional theory (DFT) calculations were carried out using the Vienna *Ab initio* Simulation Package (VASP, version 6.4.2),[7-10] employing the Gamma point for Brillouin zone sampling. This approach is appropriate for large supercell models or systems with limited periodicity. The exchange–correlation effects were treated using the Perdew-Burke-Ernzerhof (PBE) functional[11] within the generalised gradient approximation (GGA). A plane-wave energy cutoff of 415 eV was used to ensure adequate basis set convergence. Long-range dispersion interactions were accounted for using the DFT-D3 method with Becke–Johnson damping.[12] All calculations were carried out in a spin-unpolarised framework. Geometry optimisations were performed using the conjugate gradient algorithm. Electronic self-consistency was achieved when the total energy change between successive steps was less than 0.01 meV, while structural relaxation was considered converged when the residual forces on atoms were below 0.01 eV/Å.

This original ***p*MOP** structure was non-porous and yielded negligible CO_2 uptake (see Figs. S12 and S13). In an attempt to describe the uptake observed experimentally, we followed two approaches, as described in the main paper: a) rotation of the ethoxy- groups; b) expansion of the unit cell vectors of the structure. In both cases, each resulting structure was reoptimized after the perturbation was applied, again keeping unit cell vectors fixed but optimizing all atomic positions. The same level of theory and details described above were used.

To obtain our "rotated" ***p*MOP** structures, each ethoxy- group in the unit cell was rotated around the C_{ethoxy}-C_{ring} bond in increments of ~90°. We generated 12 models in this way, corresponding to different combinations of ethoxy- group rotation, with the ethoxy- groups oriented normal or planar to the aromatic ring, which were labelled "Rotated_X". Some of these structures led to significant atomic overlap and their DFT optimisations either did not converge or converged to a very large energy value; those structures were discarded from further analysis. We were left with six stable rotated structures after DFT optimisation, which were used in GCMC calculations (see below). To obtain our "expanded" ***p*MOP** structures, we manually expanded all unit cell vectors by a fixed scaling factor, corresponding to 2, 4, 6 and 8% expansion. These were labelled "Expanded 1.0x", where "x" corresponds to the expansion level. Each of those structures was DFT optimised, again keeping unit cell vectors fixed and at the same level of theory as described above. For each expanded structure, we monitored the bond lengths of Cu-paddlewheel motifs as well as bond orders.

Geometric porosity analysis with PoreBlazer

To assess the porosity of each ***p*MOP** structure (original, rotated and expanded), their respective geometric pore volumes were calculated using PoreBlazer software (v4.0).[13] The input structure was converted from .cif to .xyz format for input into PoreBlazer. The default UFF parameters,[14] as provided in the default.dat file in the software distribution, were used for the framework atoms. A nitrogen probe atom was employed to calculate the geometrical pore volume, as described in detail in the software manual. Each model's dimensions (i.e., side lengths and yz, xz, and xy angles) were defined in the input.dat file, as were obtained from the corresponding lattice parameters from the DFT optimisation output files, and are reported in Table S4. The pore volume was extracted from the summary.dat file, and is also reported in Table S4.

Table S4. Lattice parameters (cell lengths and angles), geometric pore volumes (V_p_G), and largest cavity diameter (LCD) for original, rotated, and expanded ***p*MOP** structure models.

Model Structure	Lengths (Å)			Angles (◦)			V_p_G (cm^3/g)	LCD (Å)
	a	b	c	α	β	γ		
Original	14.917	16.308	19.105	76.771	86.135	73.070	0.202	2.74
Rotated_1	14.917	16.308	19.106	76.769	86.139	73.070	0.203	2.84
Rotated_2	14.917	16.308	19.106	76.771	86.135	73.070	0.203	3.04
Rotated_3	14.917	16.308	19.106	76.769	86.139	73.070	0.201	3.00
Rotated_4	14.917	16.308	19.106	76.769	86.139	73.070	0.202	2.71
Rotated_7	14.917	16.308	19.106	76.769	86.139	73.070	0.203	2.87
Rotated_10	14.917	16.308	19.106	76.769	86.139	73.070	0.204	2.89
Expanded_1.02	15.215	16.634	19.488	76.771	86.135	73.070	0.242	2.91
Expanded_1.04	15.513	16.960	19.870	76.771	86.135	73.070	0.284	3.13
Expanded_1.06	15.812	17.286	20.252	76.771	86.135	73.070	0.318	3.23
Expanded_1.08	16.110	17.612	20.634	76.771	86.135	73.070	0.376	3.77

Grand Canonical Monte Carlo simulations of adsorption isotherms

Grand Canonical Monte Carlo (GCMC) simulations were performed using RASPA 2.0.47 (open-source software) to compute adsorption isotherms.[15] Each simulation started from a .cif input file obtained from the DFT optimisations described above. 1×10^4 initialisation cycles and 2×10^4 sampling cycles were used for all models at a temperature of 195 K and several pressures within the relevant experimental range (up to a maximum of 50 kPa in some cases). Translation, rotation, and swap probabilities were weighed at a 1:1:2 ratio, and MC trials were either accepted or rejected based on a probability proportional to their Boltzmann factor. In all simulations, the ***p*MOP** framework was treated as a rigid structure and interactions were described by a combination of Lennard-Jones (LJ) repulsion-dispersion and Coulomb electrostatics. The CO_2 adsorbate was described using the TraPPE model,[16] while the framework LJ sites were assigned parameters from the Universal Force Field (UFF).[14] For completeness, all LJ parameters are reported in Table S5. Lorentz–Berthelot combining rules were applied to calculate cross-species LJ interactions. LJ interactions were

truncated at a cut-off radius of 12.0 Å and tail corrections were applied, since it has been shown that this eliminates the dependence of adsorption uptakes on the cut-off radius.[17-19] Partial atomic charges were calculated for each framework atom by applying the DDEC6 method to the output of the DFT optimisation of the original ***p*MOP** structure.[20] The Chargemol code was used to obtain those point charges from the output of VASP (files generated with keywords LCHARG= .TRUE. and LAECHG= .TRUE.).[21] The same charges were used for all modified structures, rotated or expanded. Although, strictly speaking, this is an approximation, we carried out tests and observed that the effect of such small structural displacements on the resulting point charges was very minor. All the simulation input files, including assigned point charges, are made openly available through the Strathclyde data repository (see link in the main paper). It is important to note that the model employed here, based only on LJ dispersion-repulsion and Coulomb electrostatics, cannot accurately describe coordination-type interactions between the CO_2 molecule and unsaturated Cu sites present in the activated ***p*MOP** structures. Accurately accounting for such interactions would require development of a bespoke QM-derived correction,[22] which is beyond the scope of this work. Because the concentration of open metal sites in ***p*MOP** is relatively low, we expect this limitation of the model to lead to a slight underestimation of the adsorbed amounts, which may nevertheless explain minor deviations between simulated and experimental isotherms, as discussed in the main paper.

Table S5. Lennard-Jones parameters for all atoms of the ***p*MOP** structure and the CO_2 molecule.

**Atom**	$\boldsymbol{\varepsilon/kB}$ (K)	$\boldsymbol{\sigma}$ (Å)
O	30.218	3.118
C	52.838	3.431
H	22.142	2.571
Cu	2.516	3.114
O_CO_2	79.0	3.05
C_CO_2	27.0	2.08

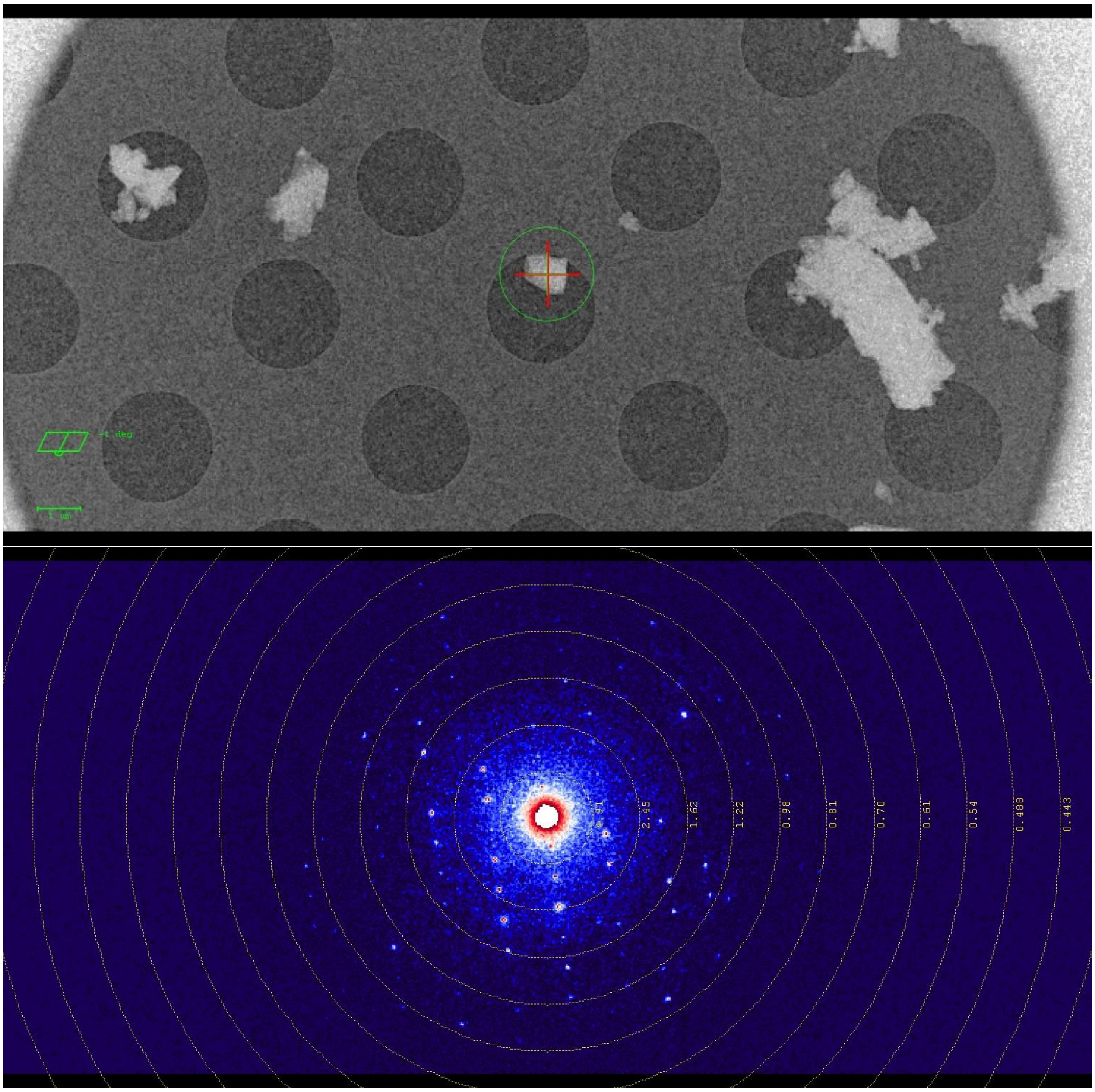

Figure S1. (Top) View of one of the crystallites used in data collection for ***p*MOP$_{H2O}$**. The scale bar at the bottom left of the image measures 1 µm. (Bottom) Diffraction image corresponding to this crystallite.

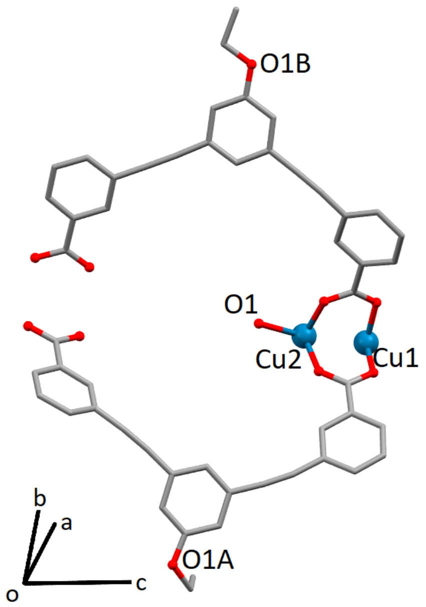

Figure S2. View of the asymmetric unit in ***p*MOP$_{H2O}$**. Hydrogen atoms have been omitted for clarity, and only heteroatoms relevant to the discussion in the main text have been labelled.

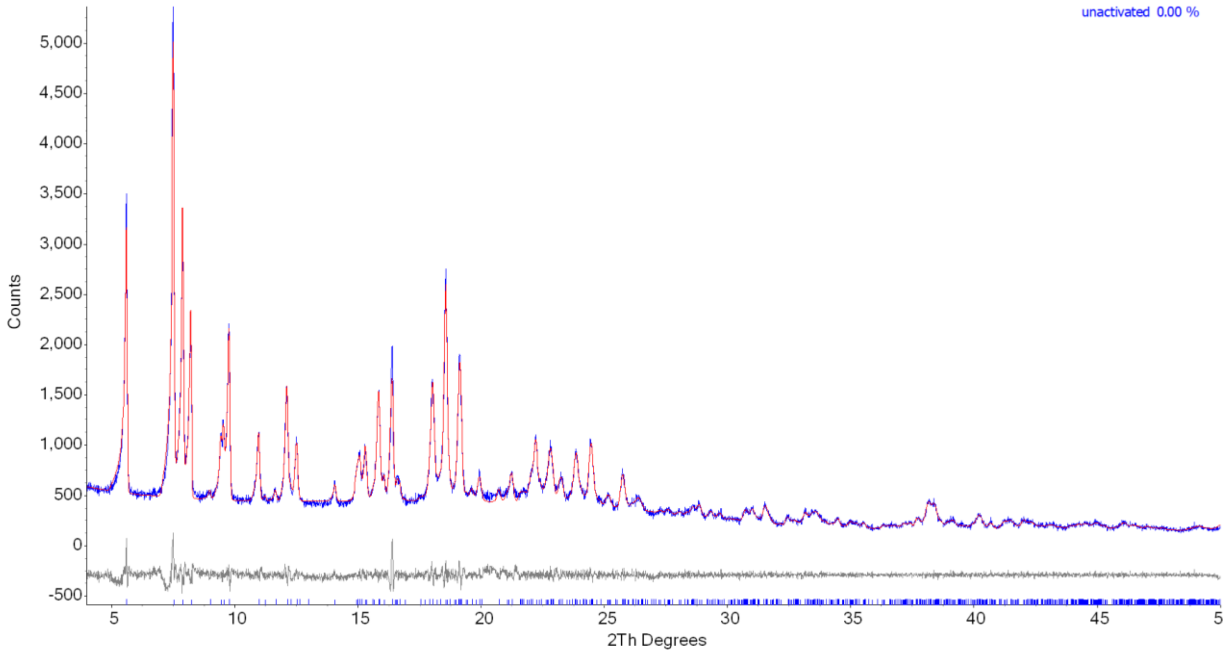

Figure S3. Pawley refinement of ***p*MOP$_{H2O}$**. Unit cell parameters: a = 12.1637(13) Å; b = 12.2440(13) Å; c = 16.6960(18) Å; α = 80.323(8)°; β = 70.848(6)°; γ = 74.166(9)°. R_{wp} = 5.84%.

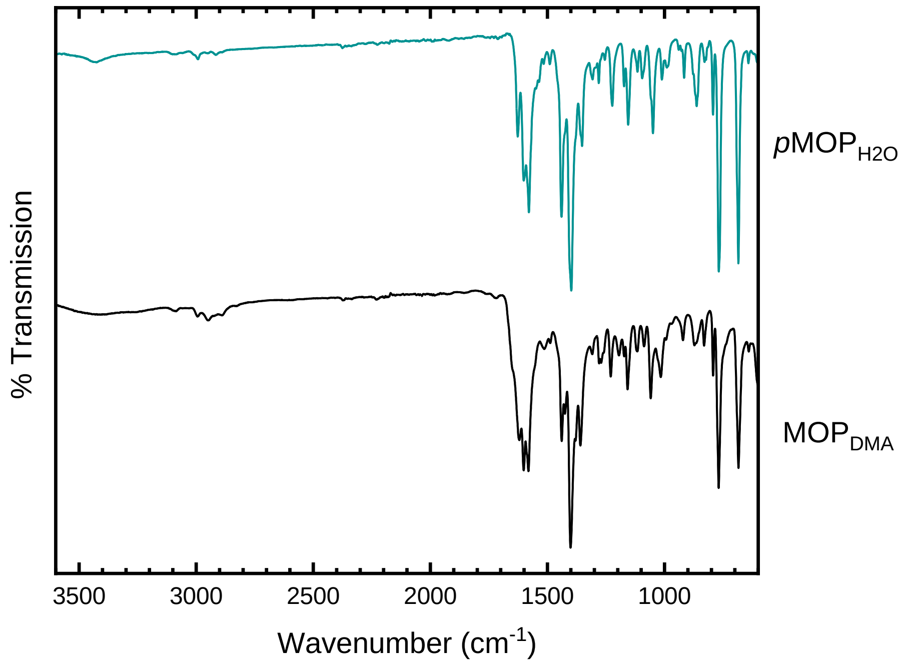

Figure S4. Comparison of the IR spectra of **MOP$_{DMA}$** and ***p*MOP$_{H2O}$**.

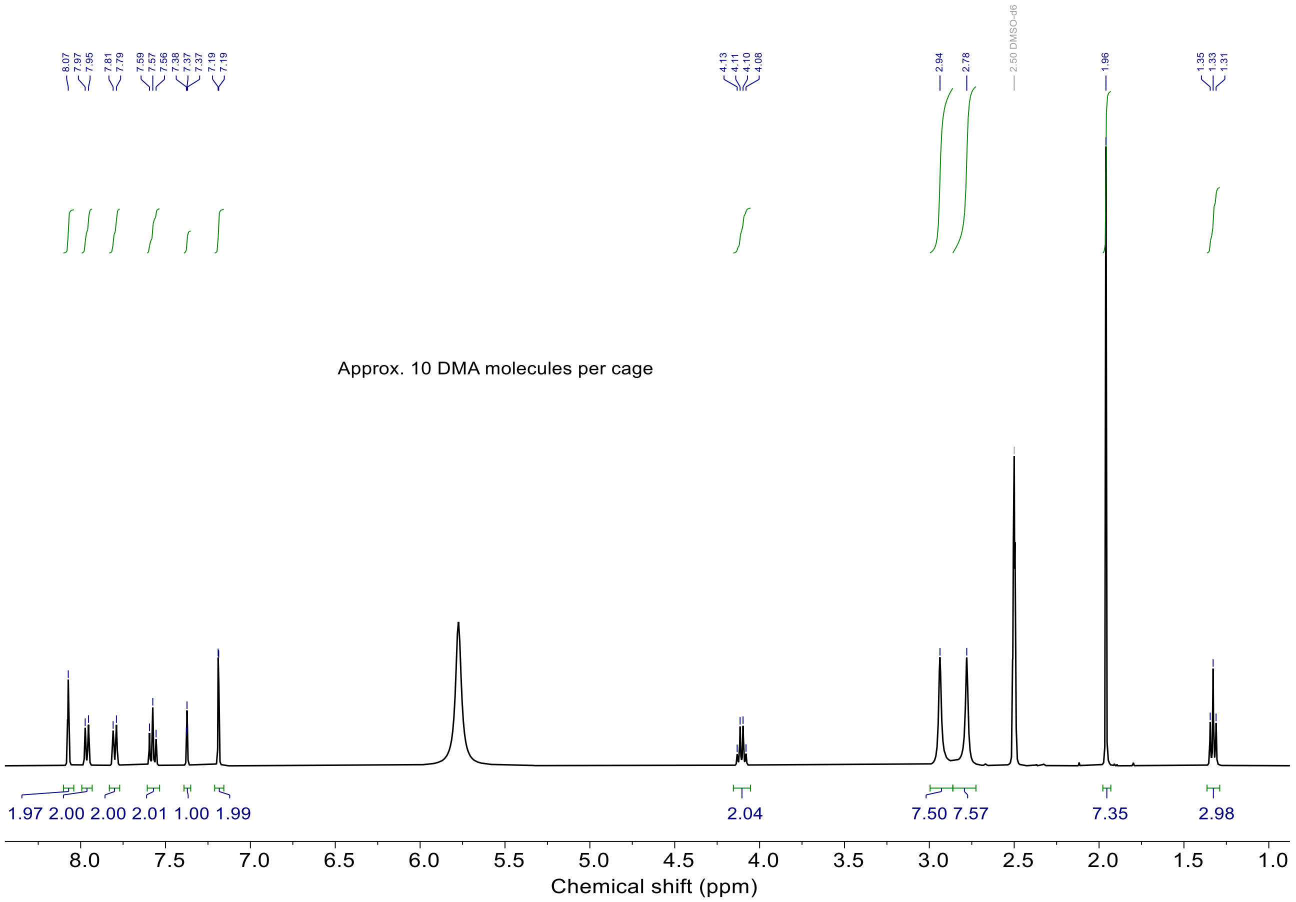

Figure S5. ^{1}H NMR spectrum run on an acid-digested sample of **MOP$_{DMA}$**.

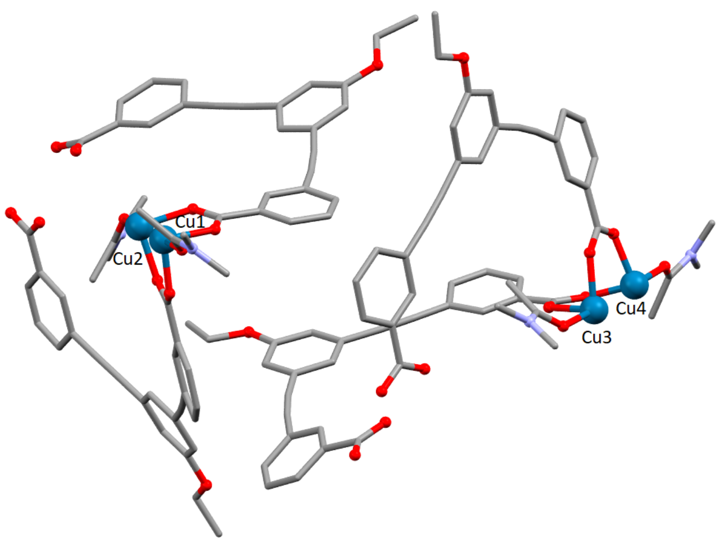

Figure S6. View of the asymmetric unit in **MOP_{DMA}**, which consists of two half lantern cages, where each cage sits on an inversion centre to complete the molecules. Hydrogen atoms, non-coordinated solvents, and disordered moieties have been omitted for clarity.

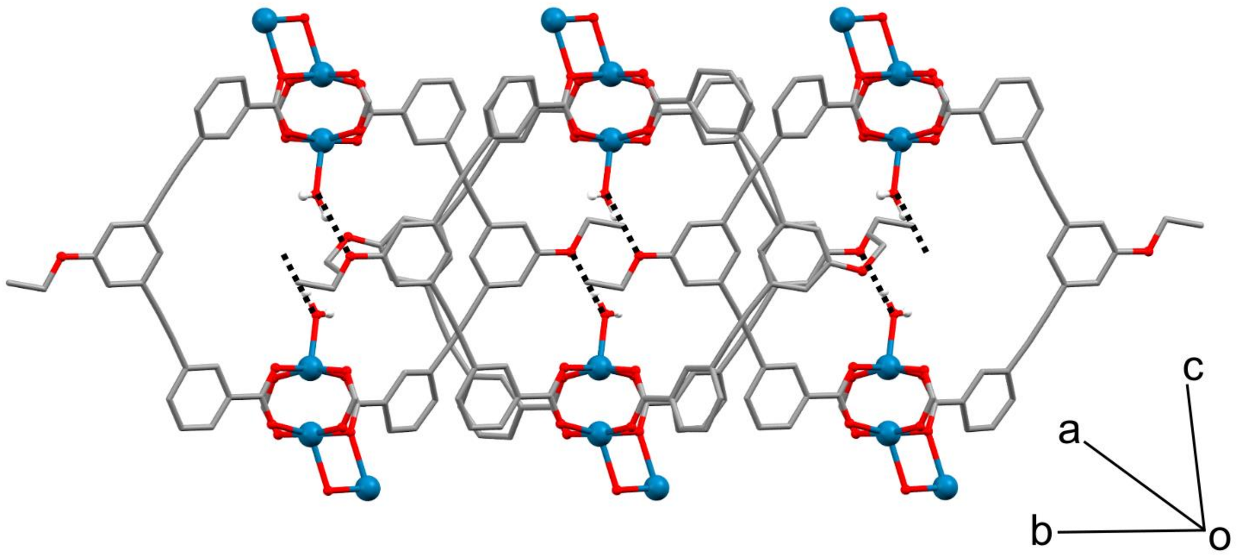

Figure S7. View of the hydrogen bonding interactions, represented as dashed lines, that form between the ethoxy- groups of neighbouring polymers and the water molecules coordinated to the inner axial sites of the Cu(II) paddlewheels ($O1_{H2O}\cdots O1B_{ethoxy}$ = 2.788(11) Å).

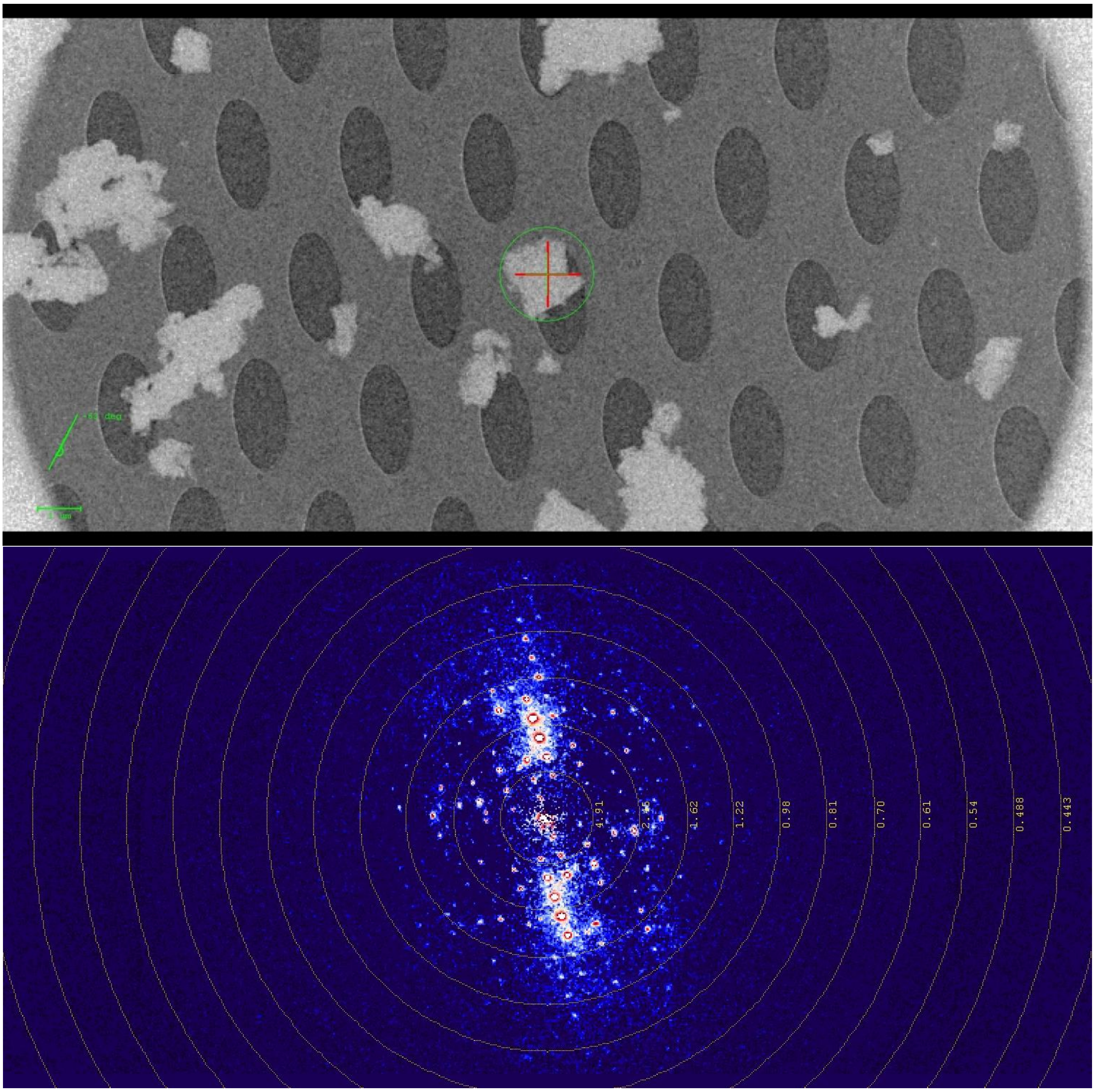

Figure S8. (Top) View of one of the crystallites used in data collection for ***p*MOP**. The scale bar at the bottom left of the image measures 1 µm. (Bottom) Diffraction image corresponding to this crystallite.

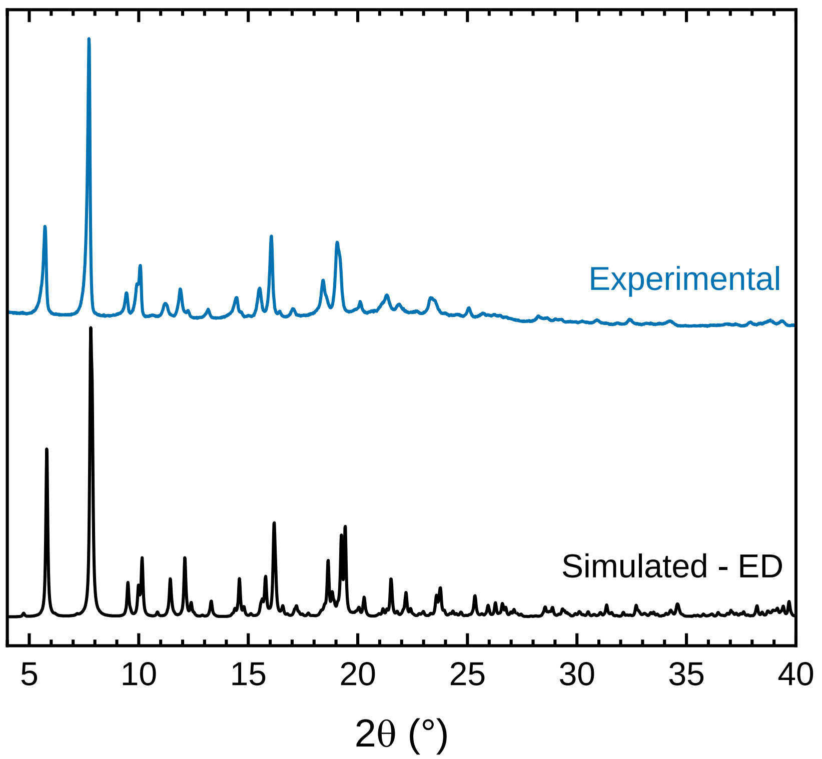

Figure S9. Comparison of the experimentally obtained PXRD data for ***p*MOP** with the simulated diffractogram generated from the .cif of the 3D ED structure.

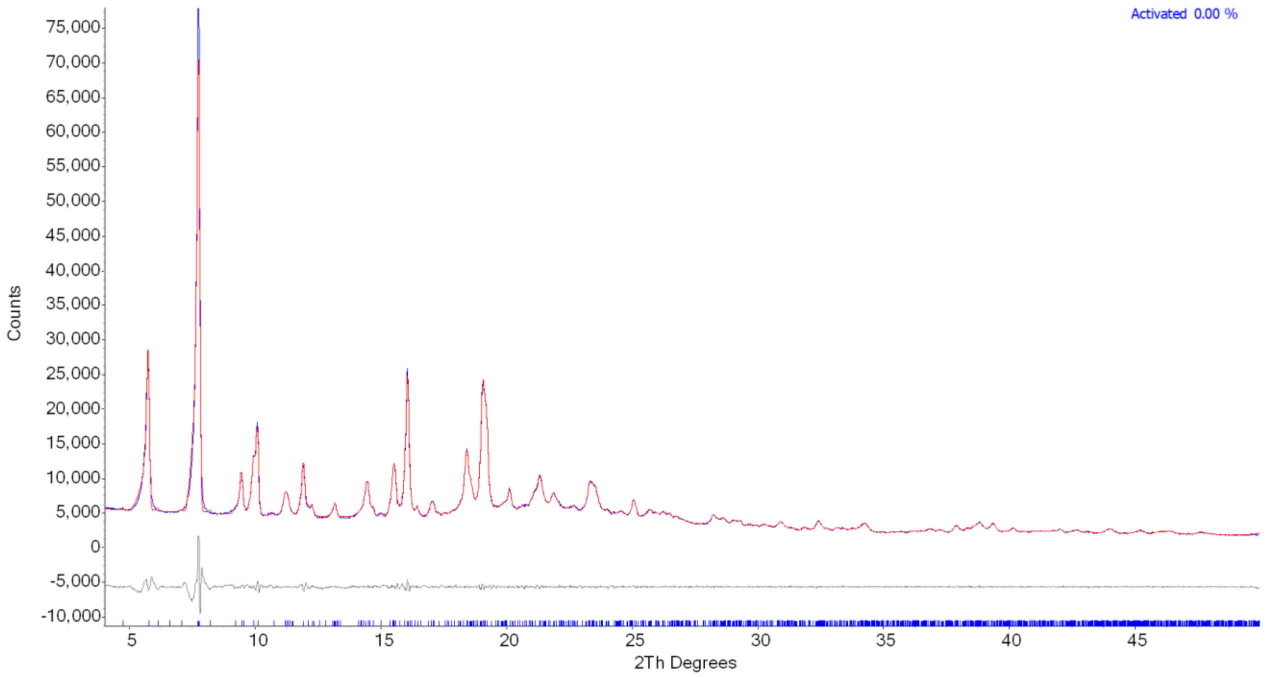

Figure S10. Pawley refinement of ***p*MOP**. Unit cell parameters: a = 15.121(7) Å; b = 16.500(14) Å; c = 19.209(10) Å; α = 76.70(4)°; β = 85.87(4)°; γ = 72.60(2)°. R_{wp} = 3.69%.

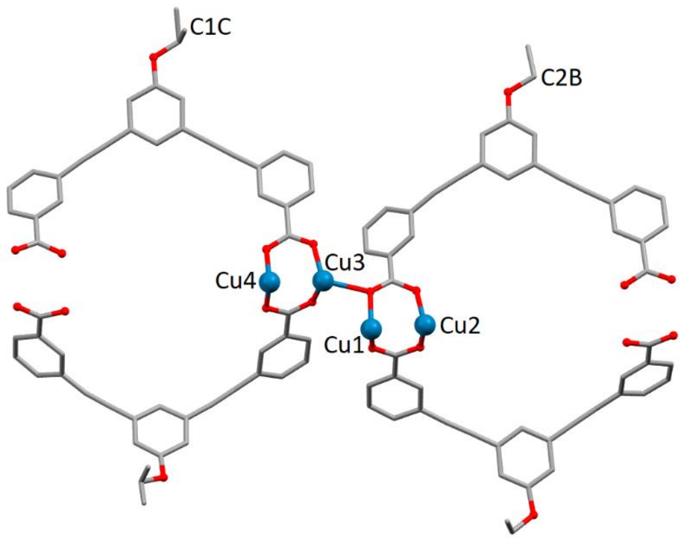

Figure S11. View of the asymmetric unit in ***p*MOP**. Hydrogen atoms have been omitted for clarity, and only heteroatoms relevant to the discussion in the main text have been labelled.

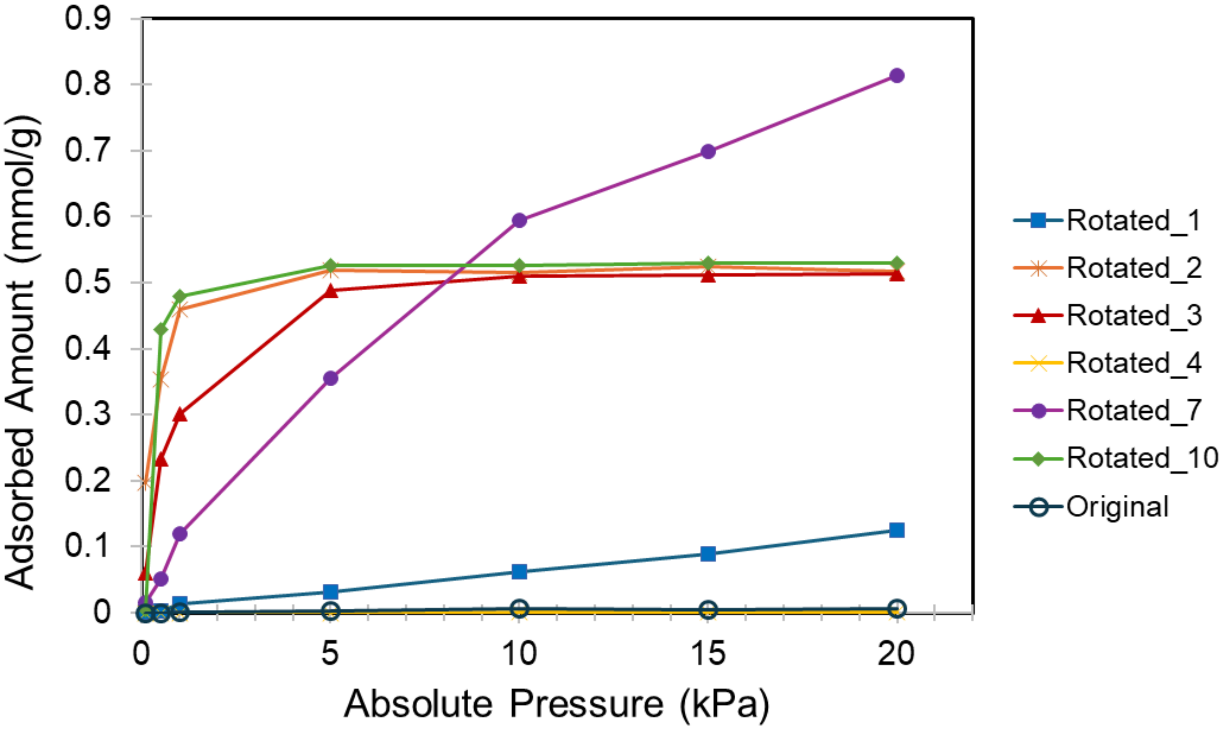

Figure S12. GCMC simulated adsorption isotherms for CO_2 at 195 K on the ***p*MOP** activated structure ("Original") and on structures with different degrees of rotation of the ethoxy- groups, after DFT optimization (the .cif files for all rotated structures, after DFT optimisation, are provided in the data repository linked in the manuscript). The observed uptake depends strongly on the ethoxy- group configuration, with some structures (including the original 3D ED structure) yielding very low or negligible adsorption, and others leading to substantial uptake with a Type I isotherm shape. While the geometric pore volumes for each structure are very similar, the adsorption uptake roughly follows the trend of increasing largest cavity diameter (see Table S4). In the main paper, Figure 5a, we show the results for model Rotated_3.

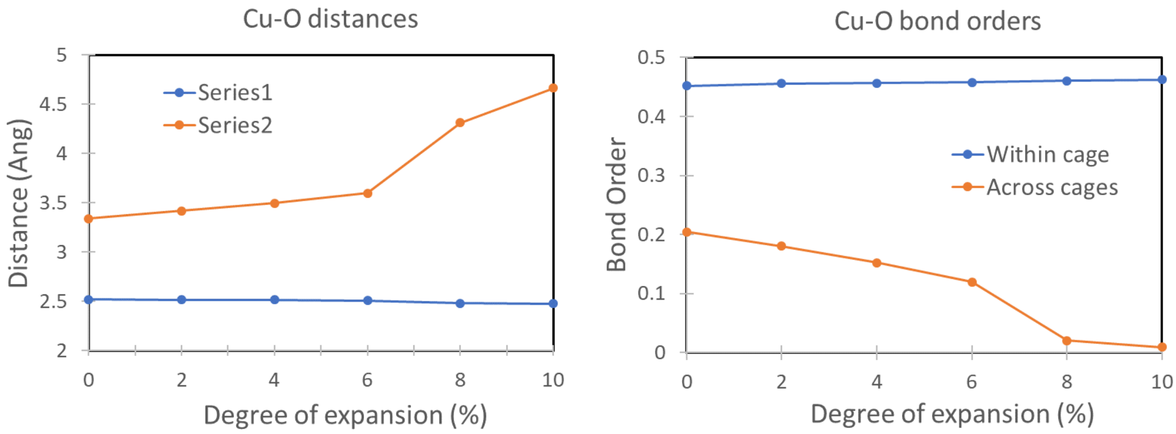

Figure S13. Bond distances (left) and bond order parameters (right) for coordination bonds between Cu atoms and oxygen atoms belonging to carboxylate groups, as calculated by DFT. The blue line refers to bonds within the same lantern-shaped cage, while the orange line refers to bonds across different cages – i.e. those that form the polymeric ***p*MOP** structure. In both cases, an abrupt shift is observed between 6 and 8% expansion, indicating rupture of the polymeric links.

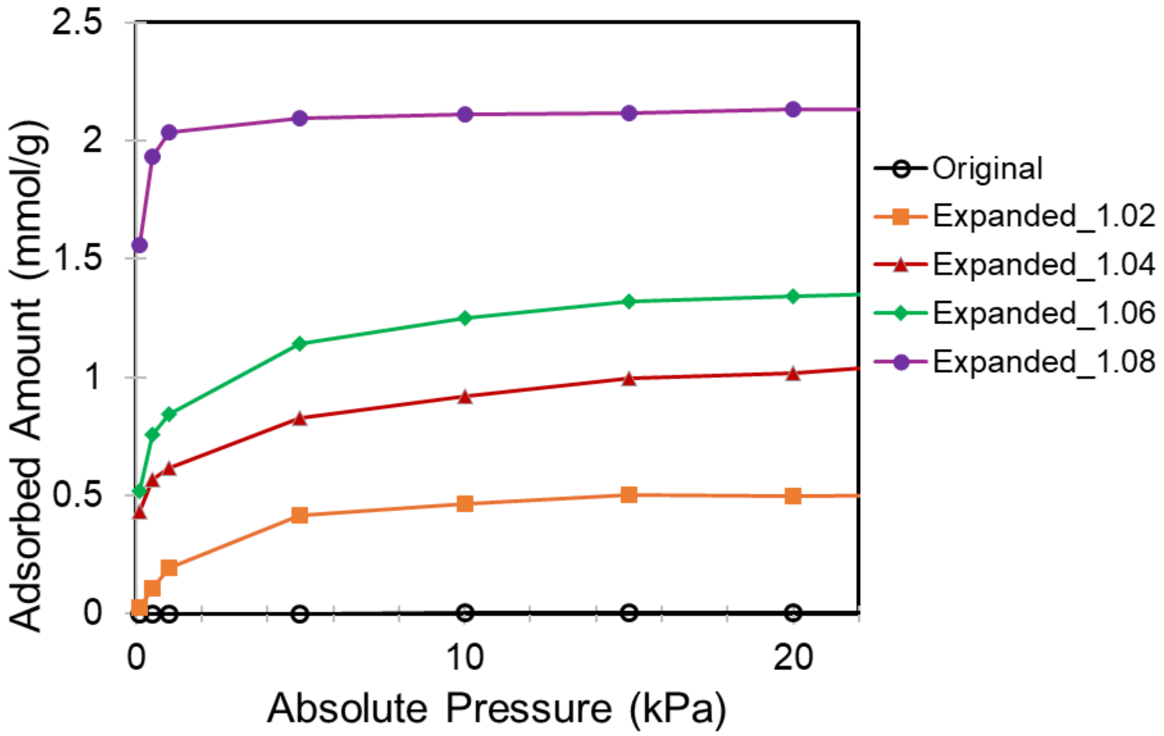

Figure S14. Simulated adsorption isotherms for CO_2 at 195 K on the ***p*MOP** activated structure ("Original") and on structures with different degrees of unit cell expansion, after DFT optimization. The adsorption uptake follows the trend of increasing accessible pore volume (Table S4). Notice also the abrupt change between 6 and 8% expansion, in line with observations in Figure S13.

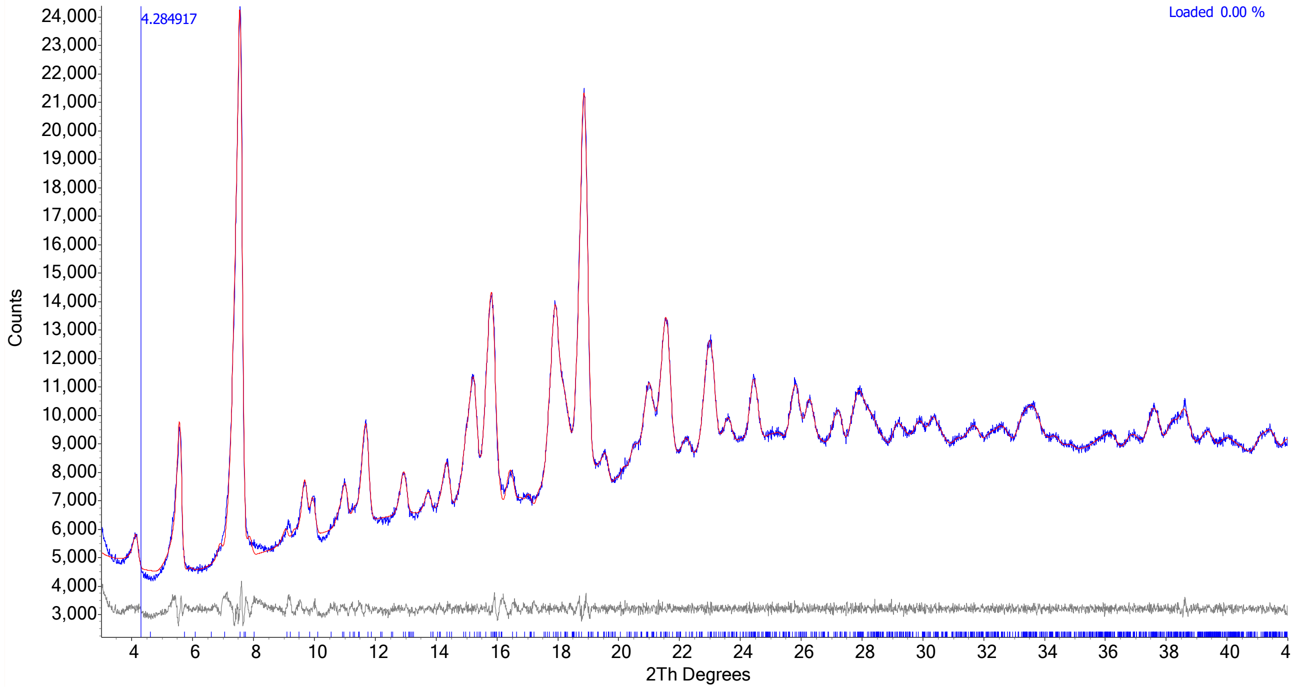

Figure S15. Pawley refinement of ***p*MOP** loaded with 17 kPa of CO_2 at 195 K. Unit cell parameters: a = 15.228(18) Å; b = 16.52(3) Å; c = 19.67(2) Å; α = 77.68(6)°; β = 85.06(7)°; γ = 72.60(4)°. R_{wp} = 1.65%. The peak at 2ϑ = 4° does not fit the cell and is modelled as a single peak. Note that the unit cell parameters for the activated unit cell, collected at 150 K, are a = 14.935 Å; b = 16.295 Å; c = 19.062 Å; α = 76.68°; β = 86.25°; γ = 73.10°, confirming the expansion of the unit cell as gas is captured, and justifying the computational approach.

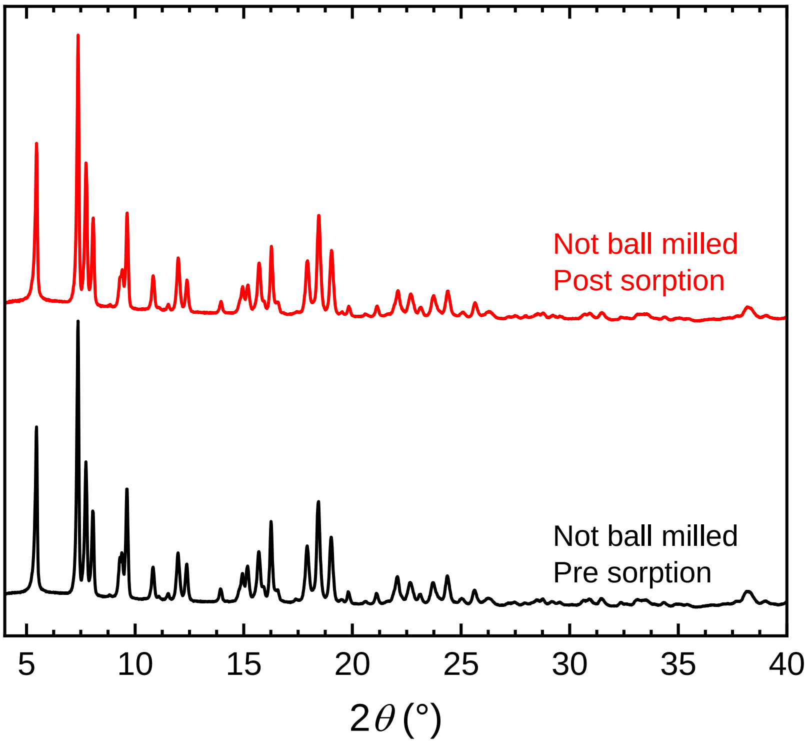

Figure S16. Comparison of the PXRD data for ***p*MOP$_{H2O}$** before and after gas sorption measurements.

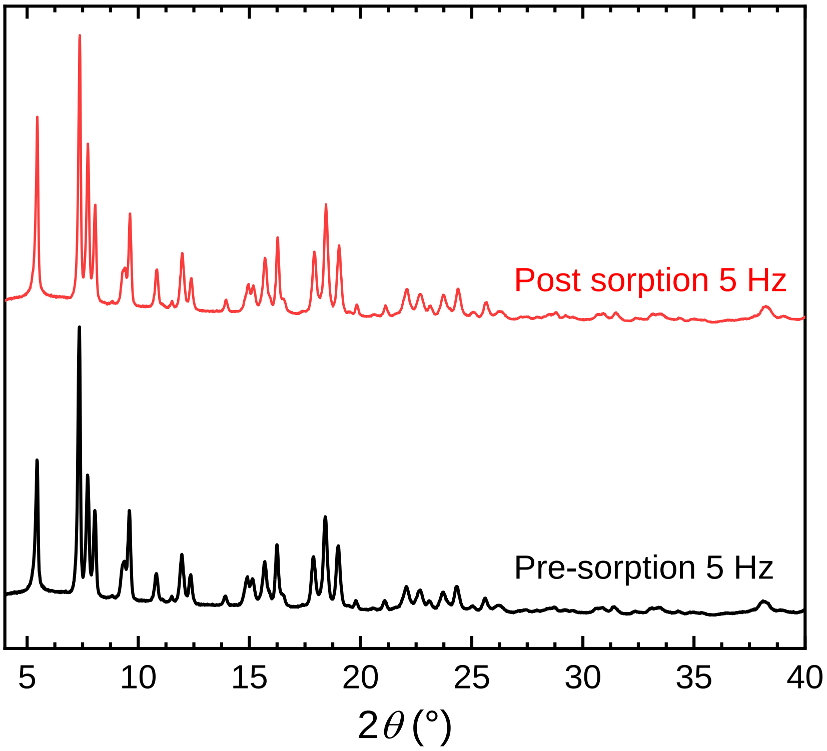

Figure S17. Comparison of the PXRD data for ***p*MOP$_{H2O}$** after ball milling at 5 Hz, before and after gas sorption measurements.

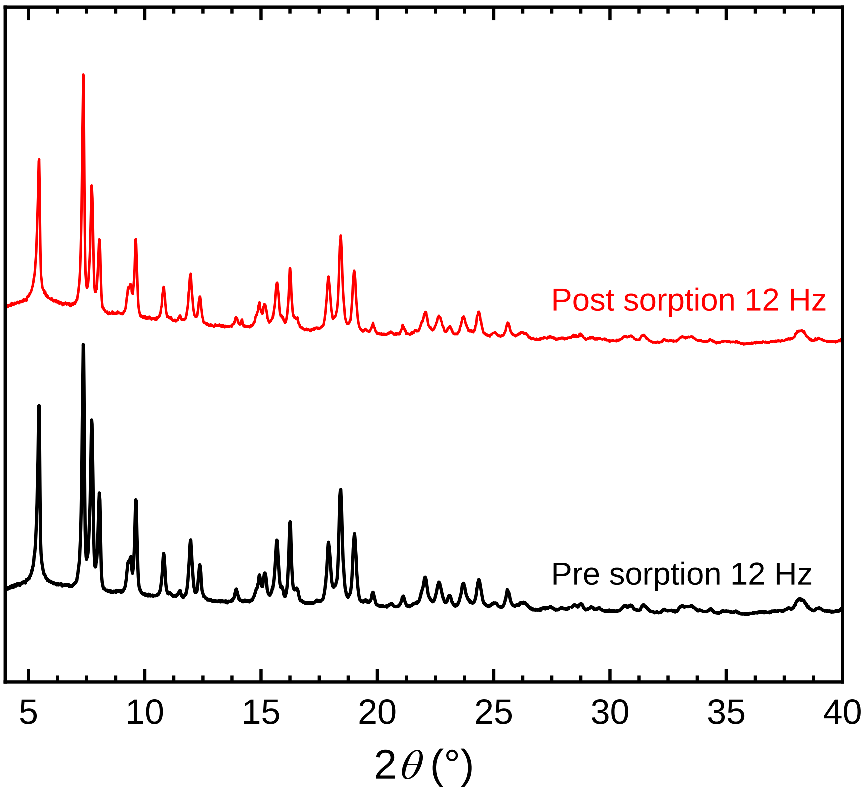

Figure S18. Comparison of the PXRD data for ***p*MOP$_{H2O}$** after ball milling at 12 Hz, before and after gas sorption measurements.

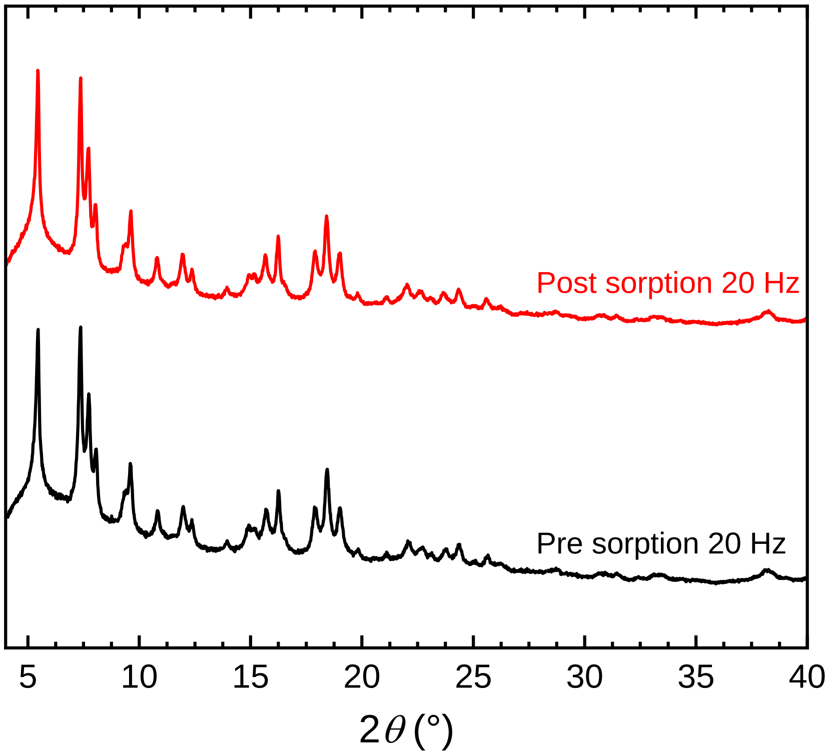

Figure S19. Comparison of the PXRD data for ***p*MOP$_{H2O}$** after ball milling at 20 Hz, before and after gas sorption measurements.

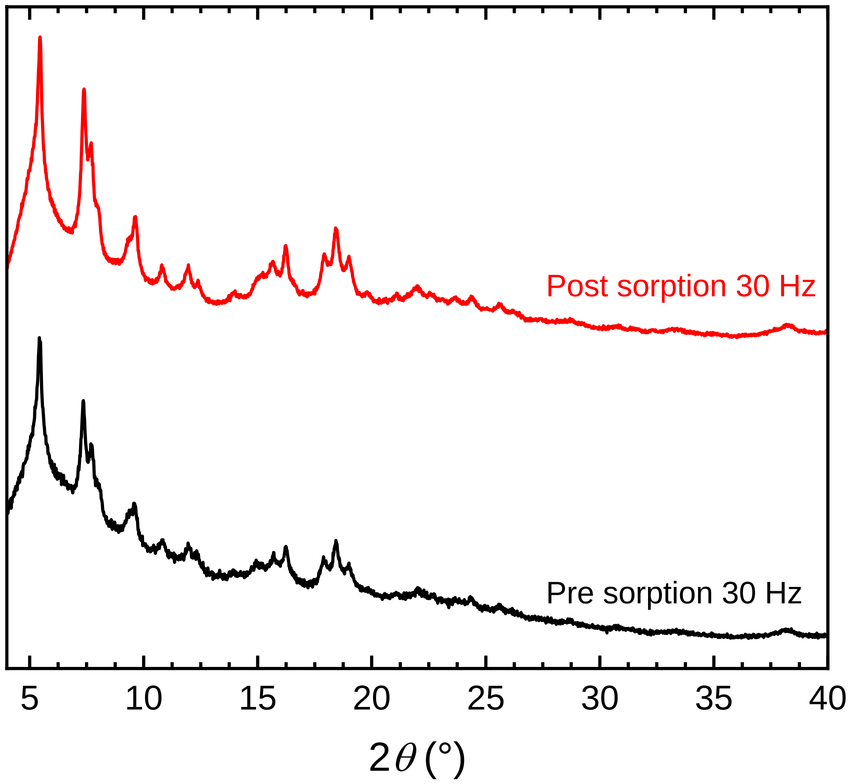

Figure S20. Comparison of the PXRD data for ***p*MOP$_{H2O}$** after ball milling at 30 Hz, before and after gas sorption measurements.

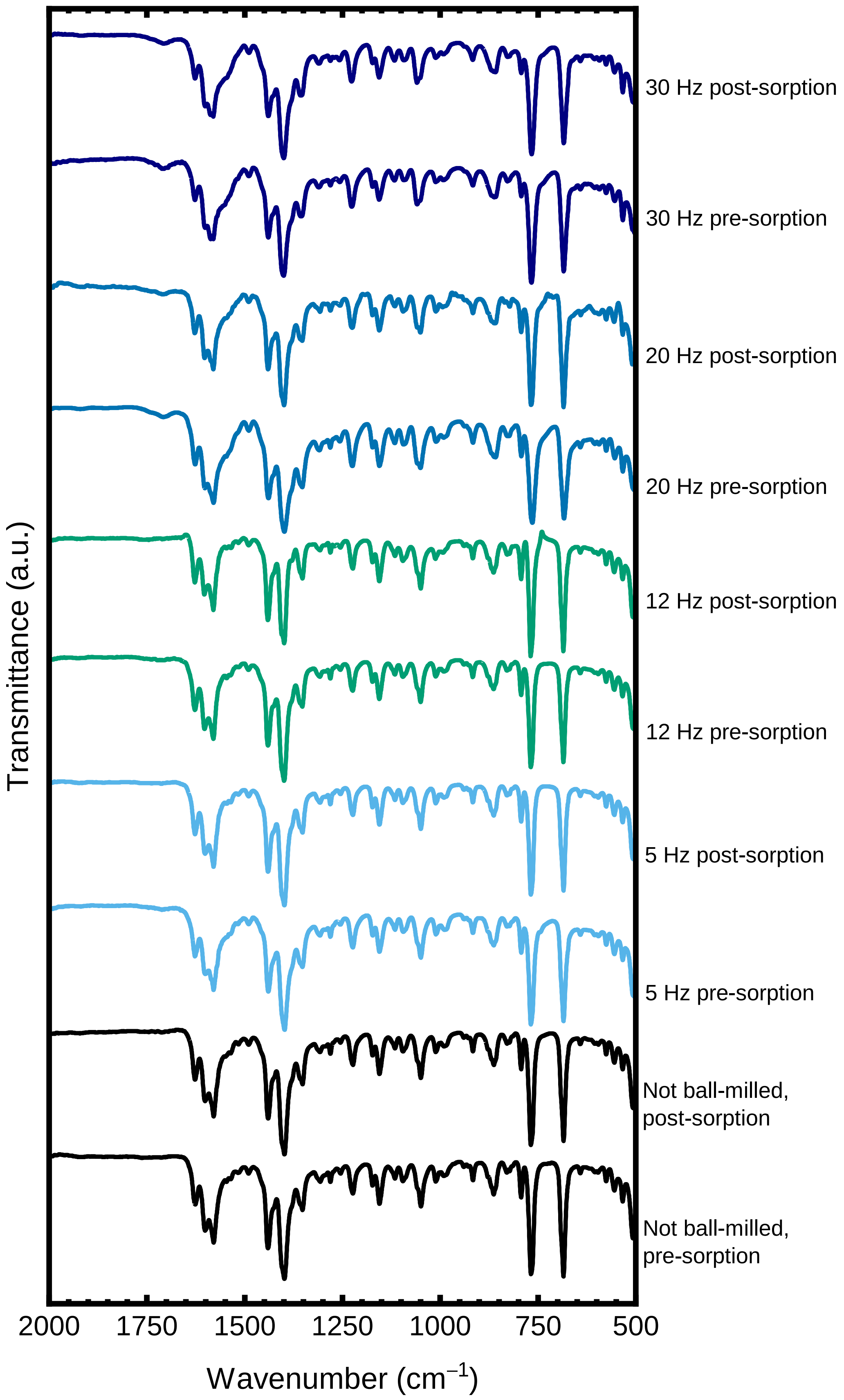

Figure S21. IR spectra for ***p*MOP$_{H2O}$** before and after gas sorption measurements, as indicated.

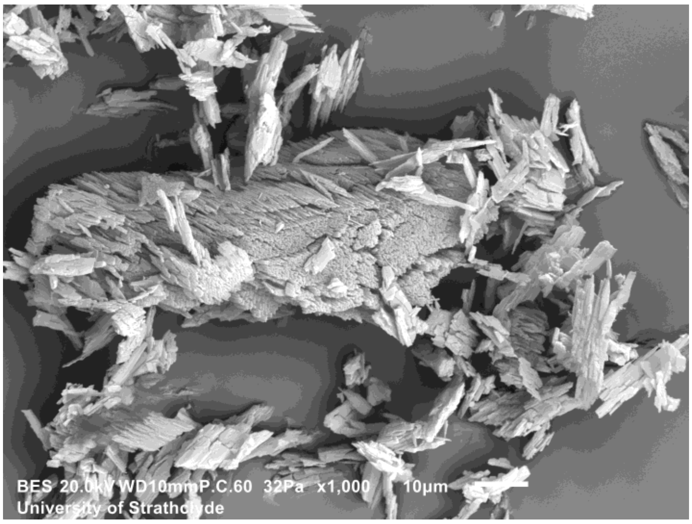

Figure S22. SEM image of the non-ball milled sample of ***p*MOP**$_{\mathbf{H2O}}$ subsequent to gas sorption measurements.

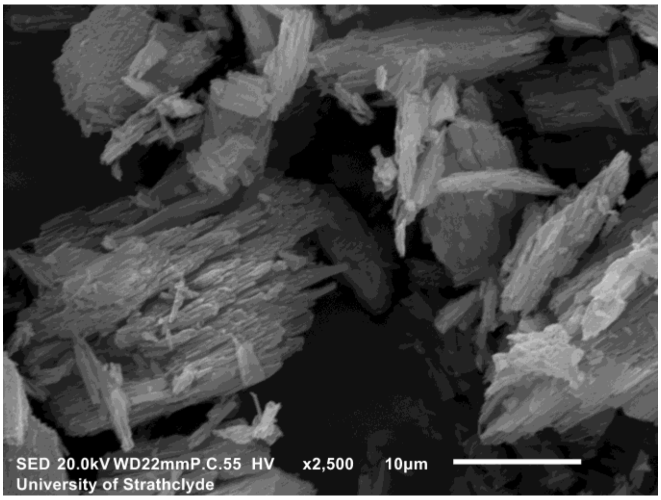

Figure S23. SEM image of the sample of ***p*MOP**$_{\mathbf{H2O}}$ that had been ball milled at 5 Hz, prior to gas sorption measurements.

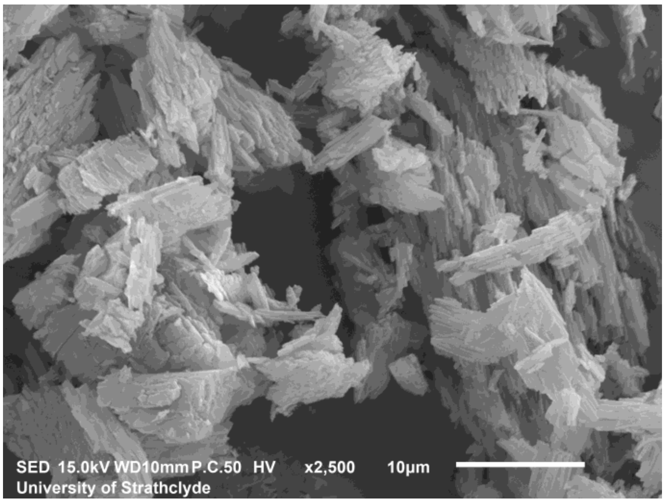

Figure S24. SEM image of the sample of ***p*MOP**$_{\mathbf{H2O}}$ that had been ball milled at 5 Hz, subsequent to gas sorption measurements.

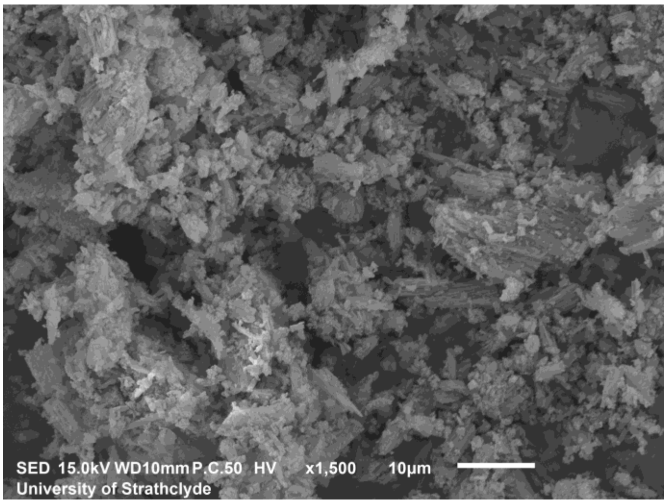

Figure S25. SEM image of the sample of ***p*MOP$_{H2O}$** that had been ball milled at 12 Hz, prior to gas sorption measurements.

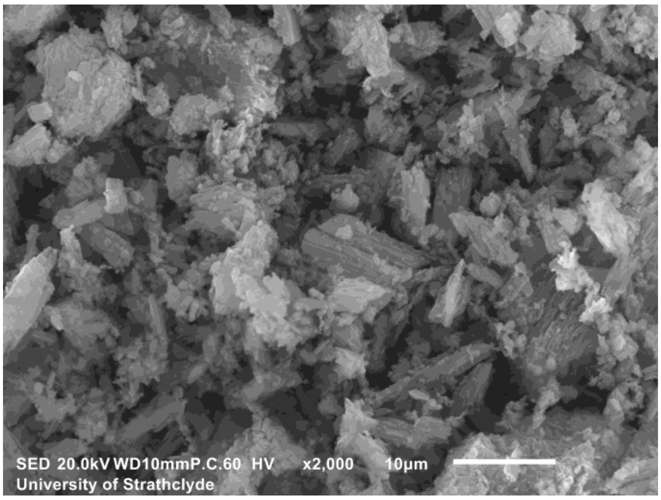

Figure S26. SEM image of the sample of ***p*MOP$_{H2O}$** that had been ball milled at 12 Hz, subsequent to gas sorption measurements.

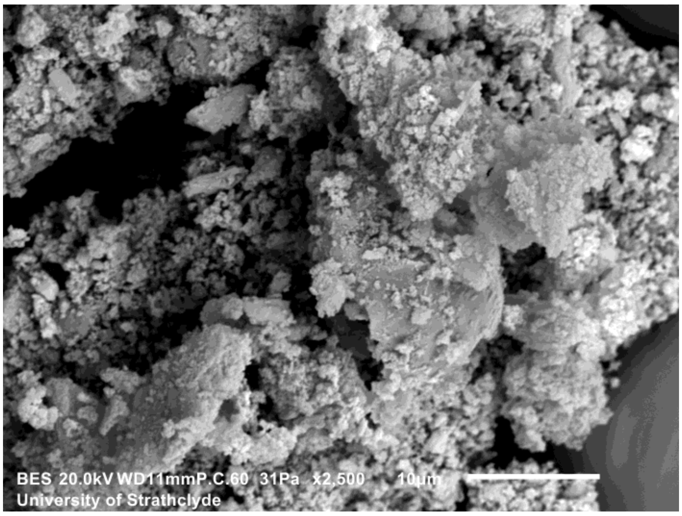

Figure S27. SEM image of the sample of ***p*MOP$_{H2O}$** that had been ball milled at 20 Hz, prior to gas sorption measurements.

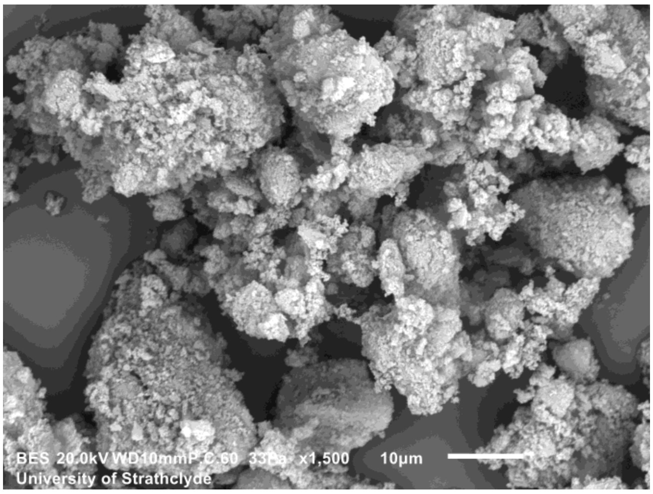

Figure S28. SEM image of the sample of ***p*MOP**$_{H2O}$ that had been ball milled at 20 Hz, subsequent to gas sorption measurements.

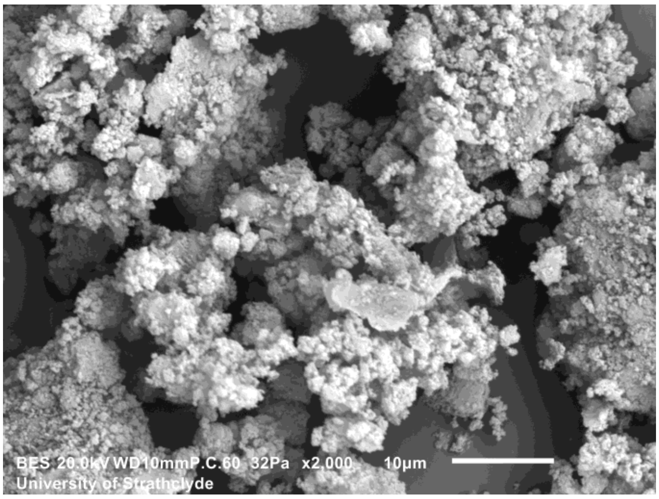

Figure S29. SEM image of the sample of ***p*MOP**$_{H2O}$ that had been ball milled at 30 Hz, prior to gas sorption measurements.

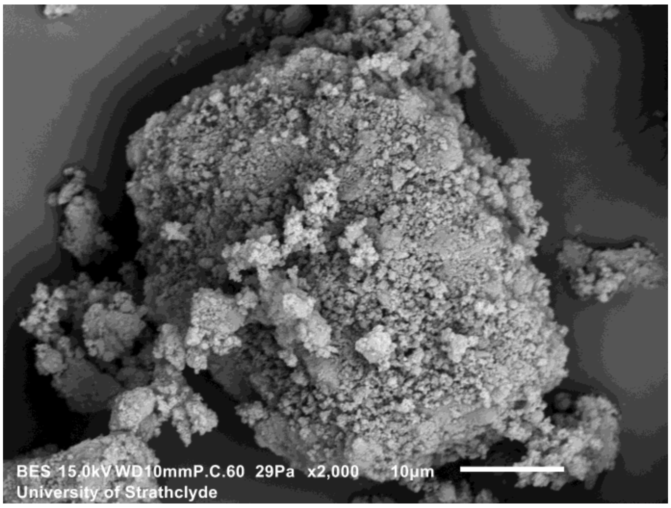

Figure S30. SEM image of the sample of ***p*MOP**$_{H2O}$ that had been ball milled at 30 Hz, subsequent to gas sorption measurements.

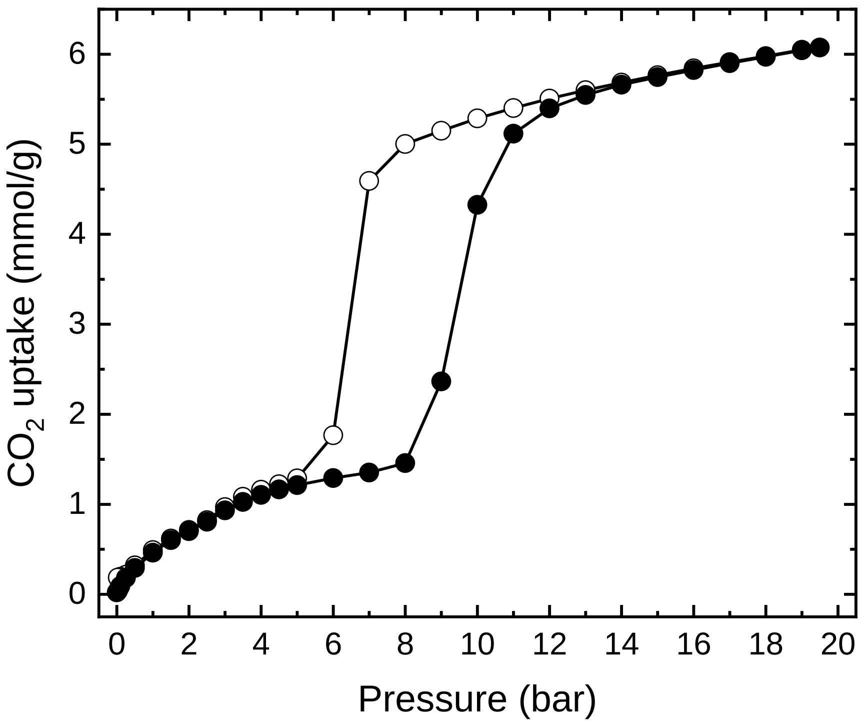

Figure S31. Full CO_2 sorption isotherm (273K) for the sample of ***p*MOP**$_{H2O}$ that had not been ball milled. Filled symbols represent adsorption, and empty symbols represent the desorption branch of the isotherm.

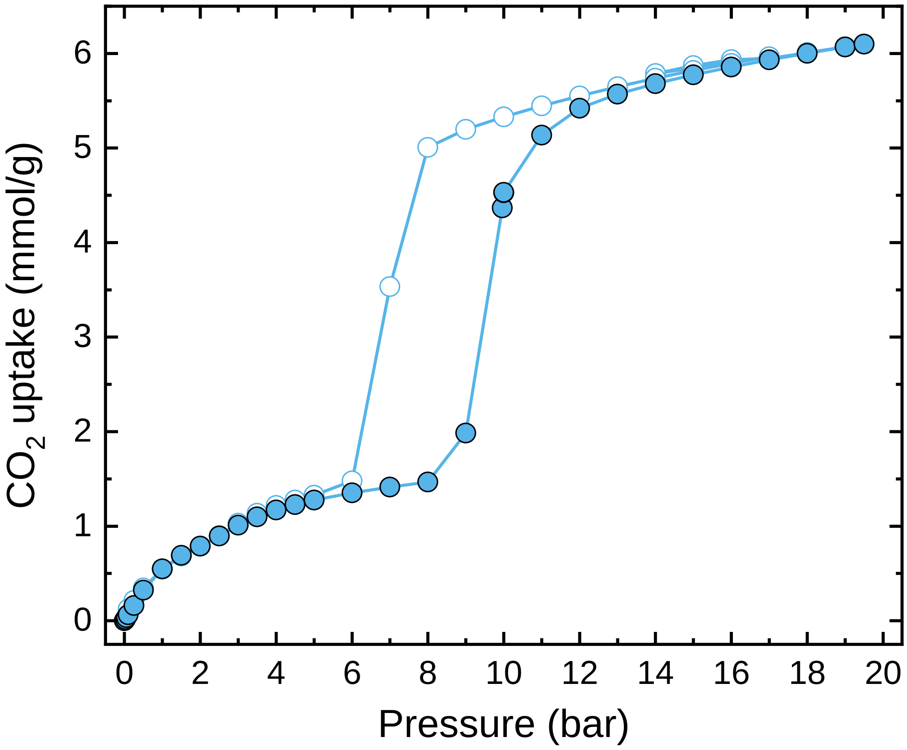

Figure S32. Full CO_2 sorption isotherm (273K) for the sample of ***p*MOP**$_{H2O}$ that had been ball milled at 5 Hz. Filled symbols represent adsorption, and empty symbols represent the desorption branch of the isotherm.

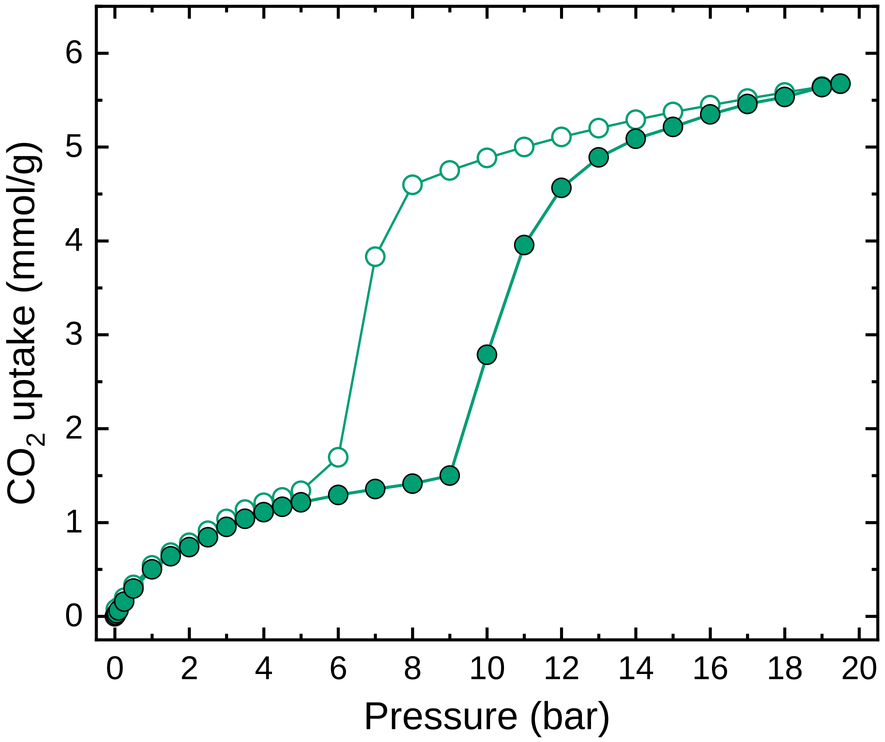

Figure S33. Full CO_2 sorption isotherm (273K) for the sample of ***p*MOP$_{H2O}$** that had been ball milled at 12 Hz. Filled symbols represent adsorption, and empty symbols represent the desorption branch of the isotherm.

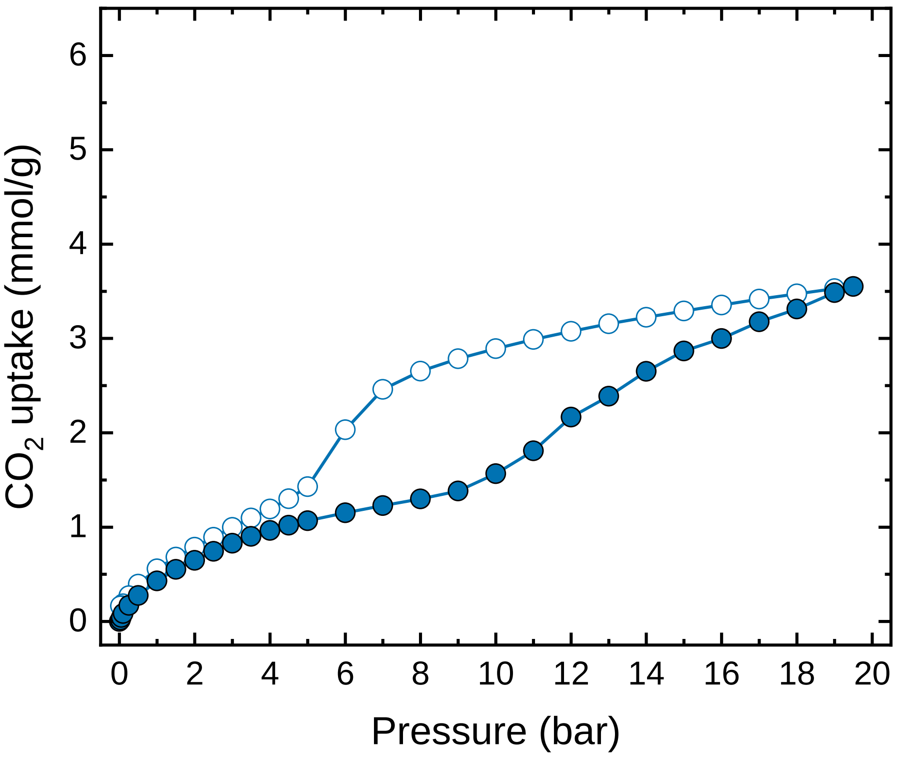

Figure S34. Full CO_2 sorption isotherm (273K) for the sample of ***p*MOP$_{H2O}$** that had been ball milled at 20 Hz. Filled symbols represent adsorption, and empty symbols represent the desorption branch of the isotherm.

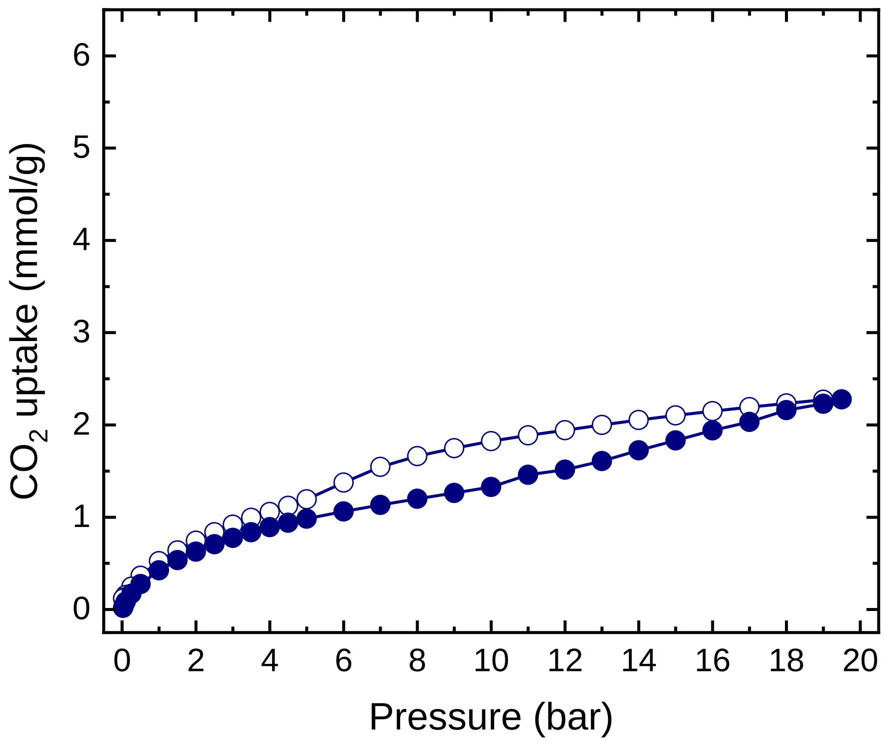

Figure S35. Full CO_2 sorption isotherm (273K) for the sample of ***p*****MOP$_{\mathbf{H2O}}$** that had been ball milled at 30 Hz. Filled symbols represent adsorption, and empty symbols represent the desorption branch of the isotherm.

References.

[1] CrysAlisPRO, Rigaku Oxford Diffraction, **2025**.

[2] G. Sheldrick, *Acta Crystallogr.* **2015**, *A71*, 3.

[3] L. J. Farrugia, *J. Appl. Cryst.* **2012**, *45*, 849.

[4] L. J. Bourhis, O. V. Dolomanov, R. J. Gildea, J. A. K. Howard, H. Puschmann, *Acta Crystallogr.* **2015**, *A71*, 59.

[5] O. V. Dolomanov, L. J. Bourhis, R. J. Gildea, J. A. K. Howard, H. Puschmann, *J. Appl. Cryst.* **2009**, *42*, 339.

[6] A. Saha, S. S. Nia, J. A. Rodríguez, *Chem. Rev.* **2022**, *122*, 13883.

[7] G. Kresse, J. Furthmüller, *Comput. Mater. Sci.* **1996**, *6*, 15.

[8] G. Kresse, J. Furthmüller, *Phys. Rev. B* **1996**, *54*, 11169.

[9] G. Kresse, J. Hafner, *Phys. Rev. B* **1994**, *49*, 14251.

[10] G. Kresse, J. Hafner, *Phys. Rev. B* **1993**, *47*, 558.

[11] J. P. Perdew, K. Burke, M. Ernzerhof, *Phys. Rev. Lett.* **1996**, *77*, 3865.

[12] S. Grimme, S. Ehrlich, L. Goerigk, *J. Comput. Chem.* **2011**, *32*, 1456.

[13] L. Sarkisov, R. Bueno-Perez, M. Sutharson, D. Fairen-Jimenez, *Chem. Mater.* **2020**, *32*, 9849.

[14] A. K. Rappe, C. J. Casewit, K. S. Colwell, W. A. Goddard, III, W. M. Skiff, *J. Am. Chem. Soc.* **1992**, *114*, 10024.

[15] D. Dubbeldam, C. Sofía, E. D. E., R. Q. and Snurr, *Molecular Simulation* **2016**, *42*, 81.

[16] J. J. Potoff, J. I. Siepmann, *AIChE J.* **2001**, *47*, 1676.

[17] S. Vandenbrande, T. Verstraelen, J. J. Gutiérrez-Sevillano, M. Waroquier, V. Van Speybroeck, *J. Phys. Chem. C* **2017**, *121*, 25309.

[18] K. M. Jablonka, D. Ongari, B. Smit, *J. Chem. Theory Comput.* **2019**, *15*, 5635.

[19] C. McCready, K. Sladekova, S. Conroy, J. R. B. Gomes, A. J. Fletcher, M. Jorge, *J. Chem. Theory Comput.* **2024**, *20*, 4869.

[20] T. A. Manz, N. G. Limas, *RSC Adv.* **2016**, *6*, 47771.

[21] N. G. Limas, T. A. Manz, *RSC Adv.* **2018**, *8*, 2678.

[22] C. Campbell, J. R. B. Gomes, M. Fischer, M. Jorge, *J. Phys. Chem. Lett.* **2018**, *9*, 3544.
