# Supplementary material for: A Metal‐Organic Polyhedron‐to‐Coordination Polymer Transition Revealed by 3D Electron Diffraction
Source: Angew Chem Int Ed Engl. 2025 Sep 6;64(43):e202514527. doi: 10.1002/anie.202514527 (PMC12535363; doi:10.1002/anie.202514527)
Supplement: Supplementary file 2 — Supporting information [file ANIE-64-e202514527-s001.zip › Crystallographic_files_for_upload/cifcheck.docx]

**checkCIF (basic structural check) running**

*Checking for embedded fcf data in CIF ...*
*Found embedded fcf data in CIF. Extracting fcf data from uploaded CIF, please wait***. . . . . . . . . . . . . . . . . . . .**

**checkCIF/PLATON (basic structural check)**

Structure factors have been supplied for datablock(s) MOPDMA

THIS REPORT IS FOR GUIDANCE ONLY. IF USED AS PART OF A REVIEW PROCEDURE FOR PUBLICATION, IT SHOULD NOT REPLACE THE EXPERTISE OF AN EXPERIENCED CRYSTALLOGRAPHIC REFEREE.

No syntax errors found. [CIF dictionary](https://www.iucr.org/iucr-top/cif/cif_core/definitions/index.html)
Please wait while processing .... [Interpreting this report](https://journals.iucr.org/services/cif/checking/checkcifreport.html)

[Structure factor report](https://checkcif.iucr.org/2nEd2UDZYRRHh/073025104525171933133/ckf.html)

**Datablock: MOPDMA**

| Bond precision: | C-C = 0.0126 A | Wavelength=1.54184 |
| --- | --- | --- |

| Cell: | a=17.6359(4) | b=18.0833(5) | c=27.0228(3) |
| --- | --- | --- | --- |
|  | alpha=88.497(2) | beta=89.830(1) | gamma=63.521(2) |
| Temperature: | 150 K |  |  |

|  | Calculated | Reported |
| --- | --- | --- |
| Volume | 7710.9(3) | 7710.9(3) |
| Space group | P -1 | P -1 |
| Hall group | -P 1 | -P 1 |
| Moiety formula | C120 H100 Cu4 N4 O24, 5.583(C4 H9 N O) | ? |
| Sum formula | C142.33 H150.25 Cu4 N9.58 O29.58 | C142.33 H150.25 Cu4 N9.58 O29.58 |
| Mr | 2722.67 | 2722.62 |
| Dx,g cm-3 | 1.173 | 1.173 |
| Z | 2 | 2 |
| Mu (mm-1) | 1.178 | 1.178 |
| F000 | 2848.0 | 2848.0 |
| F000' | 2840.46 |  |
| h,k,lmax | 21,22,32 | 21,22,32 |
| Nref | 29219 | 103232 |
| Tmin,Tmax | 0.844,0.868 | 0.644,1.000 |
| Tmin' | 0.670 |  |

| Correction method= # Reported T Limits: Tmin=0.644 Tmax=1.000 AbsCorr = MULTI-SCAN |  |
| --- | --- |

| Data completeness= 3.533 | Theta(max)= 69.999 |
| --- | --- |

| R(reflections)= 0.1257( 73152) | wR2(reflections)= 0.3808(103232) |
| --- | --- |
| \| S = 1.399 \| Npar= 1748 \| \| --- \| --- \| |  |

The following ALERTS were generated. Each ALERT has the format

**test-name_ALERT_alert-type_alert-level**.

Click on the hyperlinks for more details of the test.


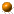
**Alert level B**

[PLAT084_ALERT_3_B](javascript:makeHelpWindow(%22PLAT084.html%22)) High wR2 Value (i.e. > 0.25) ................... 0.38 Report

AUTHOR REPLY. Given the obvious limitations of this structure (e.g. disorder, uncertainty wrt solvent sites, high R factors) this is not unusual.


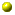
**Alert level C**

[PLAT082_ALERT_2_C](javascript:makeHelpWindow(%22PLAT082.html%22)) High R1 Value .................................. 0.13 Report

[PLAT202_ALERT_3_C](javascript:makeHelpWindow(%22PLAT202.html%22)) Isotropic non-H Atoms in Anion/Solvent ......... 5 Check

N179 C180 C182 C183 C184

[PLAT220_ALERT_2_C](javascript:makeHelpWindow(%22PLAT220.html%22)) NonSolvent Resd 1 C Ueq(max)/Ueq(min) Range 3.4 Ratio

[PLAT220_ALERT_2_C](javascript:makeHelpWindow(%22PLAT220.html%22)) NonSolvent Resd 2 C Ueq(max)/Ueq(min) Range 3.3 Ratio

[PLAT222_ALERT_3_C](javascript:makeHelpWindow(%22PLAT222.html%22)) NonSolvent Resd 1 H Uiso(max)/Uiso(min) Range 4.1 Ratio

[PLAT242_ALERT_2_C](javascript:makeHelpWindow(%22PLAT242.html%22)) Low 'MainMol' Ueq as Compared to Neighbors of N149 Check

**And 2 other PLAT242 Alerts**

More ...

[PLAT260_ALERT_2_C](javascript:makeHelpWindow(%22PLAT260.html%22)) Large Average Ueq of Residue Including O219 0.119 Check

[PLAT341_ALERT_3_C](javascript:makeHelpWindow(%22PLAT341.html%22)) Low Bond Precision on C-C Bonds ............... 0.01258 Ang.

[PLAT369_ALERT_2_C](javascript:makeHelpWindow(%22PLAT369.html%22)) Long C(sp2)-C(sp2) Bond C22A - C24A . 1.53 Ang.

[PLAT412_ALERT_2_C](javascript:makeHelpWindow(%22PLAT412.html%22)) Short Intra XH3 .. XHn H14M ..H15G . 1.82 Ang.

x,y,z = 1_555 Check

[PLAT601_ALERT_2_C](javascript:makeHelpWindow(%22PLAT601.html%22)) Unit Cell Contains Solvent Accessible VOIDS <= 77 Ang**3

[PLAT906_ALERT_3_C](javascript:makeHelpWindow(%22PLAT906.html%22)) Large K Value in the Analysis of Variance ...... 11.306 Check

[PLAT906_ALERT_3_C](javascript:makeHelpWindow(%22PLAT906.html%22)) Large K Value in the Analysis of Variance ...... 2.330 Check

[PLAT911_ALERT_3_C](javascript:makeHelpWindow(%22PLAT911.html%22)) Missing FCF Refl Between Thmin & STh/L= 0.600 51 Report

-17 3 6, -16 4 6, -15-17 7, -14-17 7, -15-16 7, -15-15 7,

-14-14 7, -2 -9 7, -11 2 7, -10 2 7, -8 4 7, -14-17 8,

-11 2 8, -10 2 8, 11 4 8, 20 12 8, 1 13 8, -15 -6 9,

-11 2 9, 20 12 9, 1 13 9, 19 10 10, 9 5 11, 10 5 11,

-18 -6 12, -11 1 12, 9 5 12, -16 -6 13, 15 14 16, 15 16 16,

( 21 More Missing: see the .ckf listing file)

[PLAT918_ALERT_3_C](javascript:makeHelpWindow(%22PLAT918.html%22)) Reflection(s) with I(obs) much Smaller I(calc) . 71 Check


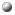
**Alert level G**

[PLAT002_ALERT_2_G](javascript:makeHelpWindow(%22PLAT002.html%22)) Number of Distance or Angle Restraints on AtSite 35 Note

[PLAT003_ALERT_2_G](javascript:makeHelpWindow(%22PLAT003.html%22)) Number of Uiso or U(i,j) Restrained non-H-Atoms 44 Report

[PLAT171_ALERT_4_G](javascript:makeHelpWindow(%22PLAT171.html%22)) The CIF-Embedded .res File Contains EADP Records 1 Report

[PLAT172_ALERT_4_G](javascript:makeHelpWindow(%22PLAT172.html%22)) The CIF-Embedded .res File Contains DFIX Records 8 Report

[PLAT174_ALERT_4_G](javascript:makeHelpWindow(%22PLAT174.html%22)) The CIF-Embedded .res File Contains FLAT Records 2 Report

[PLAT177_ALERT_4_G](javascript:makeHelpWindow(%22PLAT177.html%22)) The CIF-Embedded .res File Contains DELU Records 7 Report

[PLAT178_ALERT_4_G](javascript:makeHelpWindow(%22PLAT178.html%22)) The CIF-Embedded .res File Contains SIMU Records 7 Report

[PLAT186_ALERT_4_G](javascript:makeHelpWindow(%22PLAT186.html%22)) The CIF-Embedded .res File Contains ISOR Records 1 Report

[PLAT188_ALERT_3_G](javascript:makeHelpWindow(%22PLAT188.html%22)) A Non-default SIMU Restraint Value has been used 0.0200 Report

**And 6 other PLAT188 Alerts**

More ...

[PLAT299_ALERT_4_G](javascript:makeHelpWindow(%22PLAT299.html%22)) Atom Site Occupancy Constrained at ............. 0.5 Check

O165 N165 C164 C165 C166 C167 H16A H16B

H16C H16D H16E H16F H16J H16K H16L O175

N175 C175 C176 C177 C178 H17A H17B H17C

H17D H17E H17F H17G H17H H17I O200 N200

C201 C202 C203 C400 H20E H20F H20G H20K

H20L H20M H40A H40B H40C O201 N201 C205

C206 C207 C208 H20N H20O H20P H20Q H20R

H20S H20T H20U H20V O219 N202 C209 C220

C221 C288 H20W H22A H22B H22C H28D H28E

H28F H90X H90Z

[PLAT300_ALERT_4_G](javascript:makeHelpWindow(%22PLAT300.html%22)) Atom Site Occupancy of O174 Constrained at 0.75 Check

**And 29 other PLAT300 Alerts**

More ...

[PLAT301_ALERT_3_G](javascript:makeHelpWindow(%22PLAT301.html%22)) Main Residue Disorder ..............(Resd 1) 3% Note

[PLAT302_ALERT_4_G](javascript:makeHelpWindow(%22PLAT302.html%22)) Anion/Solvent/Minor-Residue Disorder (Resd 5) 100% Note

**And 6 other PLAT302 Alerts**

More ...

[PLAT304_ALERT_4_G](javascript:makeHelpWindow(%22PLAT304.html%22)) Non-Integer Number of Atoms in ..... (Resd 5) 7.50 Check

**And 5 other PLAT304 Alerts**

More ...

[PLAT371_ALERT_2_G](javascript:makeHelpWindow(%22PLAT371.html%22)) Long C(sp2)-C(sp1) Bond C6C - C8C . 1.42 Ang.

**And 14 other PLAT371 Alerts**

More ...

[PLAT380_ALERT_4_G](javascript:makeHelpWindow(%22PLAT380.html%22)) Incorrectly? Oriented X(sp2)-Methyl Moiety ..... C400 Check

[PLAT380_ALERT_4_G](javascript:makeHelpWindow(%22PLAT380.html%22)) Incorrectly? Oriented X(sp2)-Methyl Moiety ..... C221 Check

[PLAT413_ALERT_2_G](javascript:makeHelpWindow(%22PLAT413.html%22)) Short Inter XH3 .. XHn H14M ..H21G . 1.72 Ang.

x,y,z = 1_555 Check

[PLAT413_ALERT_2_G](javascript:makeHelpWindow(%22PLAT413.html%22)) Short Inter XH3 .. XHn H15G ..H21G . 1.98 Ang.

x,y,z = 1_555 Check

[PLAT720_ALERT_4_G](javascript:makeHelpWindow(%22PLAT720.html%22)) Number of Unusual/Non-Standard Labels .......... 16 Note

H2RG H2RH H2RI H2VG H2VH H2VI H1XX H1ZG

H1ZH H1ZI H1ZD H1ZE H1ZF H1ZB H1ZC H1QD

[PLAT790_ALERT_4_G](javascript:makeHelpWindow(%22PLAT790.html%22)) Centre of Gravity not Within Unit Cell: Resd. # 3 Note

C4 H9 N O

**And 4 other PLAT790 Alerts**

More ...

[PLAT860_ALERT_3_G](javascript:makeHelpWindow(%22PLAT860.html%22)) Number of Least-Squares Restraints ............. 329 Note

[PLAT870_ALERT_4_G](javascript:makeHelpWindow(%22PLAT870.html%22)) ALERTS Related to Twinning Effects Suppressed .. ! Info

[PLAT883_ALERT_1_G](javascript:makeHelpWindow(%22PLAT883.html%22)) Absent Datum for _atom_sites_solution_primary .. Please Do !

[PLAT899_ALERT_4_G](javascript:makeHelpWindow(%22PLAT899.html%22)) SHELXL2018 is Outdated and Succeeded by SHELXL 2019/3 Note

[PLAT910_ALERT_3_G](javascript:makeHelpWindow(%22PLAT910.html%22)) Missing FCF Reflection(s) Below Theta(Min)[Deg]= 2.73 Note

0 0 1,

[PLAT912_ALERT_4_G](javascript:makeHelpWindow(%22PLAT912.html%22)) Missing # of FCF Reflections Above STh/L= 0.600 16 Note

[PLAT931_ALERT_5_G](javascript:makeHelpWindow(%22PLAT931.html%22)) CIFcalcFCF Twin Law [ 0 0 1] Est.d BASF 0.40 Check

[PLAT933_ALERT_2_G](javascript:makeHelpWindow(%22PLAT933.html%22)) Number of HKL-OMIT Records in Embedded .res File 3 Note

-2-11 20, 3-11 17, 11 -1-12,

[PLAT941_ALERT_3_G](javascript:makeHelpWindow(%22PLAT941.html%22)) Average HKL Measurement Multiplicity ........... 3.5 Low

[PLAT967_ALERT_5_G](javascript:makeHelpWindow(%22PLAT967.html%22)) Note: Two-Theta Cutoff Value in Embedded .res .. 140.0 Degree

[PLAT969_ALERT_5_G](javascript:makeHelpWindow(%22PLAT969.html%22)) The 'Henn et al.' R-Factor-gap value ........... 3.580 Note

Predicted wR2: Based on SigI**2 10.64 or SHELX Weight 27.21

0 **ALERT level A** = Most likely a serious problem - resolve or explain

1 **ALERT level B** = A potentially serious problem, consider carefully

17 **ALERT level C** = Check. Ensure it is not caused by an omission or oversight

96 **ALERT level G** = General information/check it is not something unexpected

1 ALERT type 1 CIF construction/syntax error, inconsistent or missing data

30 ALERT type 2 Indicator that the structure model may be wrong or deficient

19 ALERT type 3 Indicator that the structure quality may be low

61 ALERT type 4 Improvement, methodology, query or suggestion

3 ALERT type 5 Informative message, check

| It is advisable to attempt to resolve as many as possible of the alerts in all categories. Often the minor alerts point to easily fixed oversights, errors and omissions in your CIF or refinement strategy, so attention to these fine details can be worthwhile. In order to resolve some of the more serious problems it may be necessary to carry out additional measurements or structure refinements. However, the purpose of your study may justify the reported deviations and the more serious of these should normally be commented upon in the discussion or experimental section of a paper or in the "special_details" fields of the CIF. checkCIF was carefully designed to identify outliers and unusual parameters, but every test has its limitations and alerts that are not important in a particular case may appear. Conversely, the absence of alerts does not guarantee there are no aspects of the results needing attention. It is up to the individual to critically assess their own results and, if necessary, seek expert advice.  **Publication of your CIF in IUCr journals**  A basic structural check has been run on your CIF. These basic checks will be run on all CIFs submitted for publication in IUCr journals (*Acta Crystallographica*, *Journal of Applied Crystallography*, *Journal of Synchrotron Radiation*); however, if you intend to submit to *Acta Crystallographica Section C* or *E* or *IUCrData*, you should make sure that [full publication checks](http://journals.iucr.org/services/cif/checking/checkform.html) are run on the final version of your CIF prior to submission.  **Publication of your CIF in other journals**  Please refer to the *Notes for Authors* of the relevant journal for any special instructions relating to CIF submission. |
| --- |
