# Supplementary material for: A Metal‐Organic Polyhedron‐to‐Coordination Polymer Transition Revealed by 3D Electron Diffraction
Source: Angew Chem Int Ed Engl. 2025 Sep 6;64(43):e202514527. doi: 10.1002/anie.202514527 (PMC12535363; doi:10.1002/anie.202514527)

# checkCIF/PLATON report

Structure factors have been supplied for datablock(s) CuEtOMOP_12Hz_pMOPH2O

THIS REPORT IS FOR GUIDANCE ONLY. IF USED AS PART OF A REVIEW PROCEDURE FOR PUBLICATION, IT SHOULD NOT REPLACE THE EXPERTISE OF AN EXPERIENCED CRYSTALLOGRAPHIC REFEREE.

No syntax errors found. CIF dictionary Interpreting this report

## Datablock: CuEtOMOP_12Hz_pMOPH2O

---

```
Bond precision:   C-C = 0.0116 A          Wavelength=0.02510

Cell:           a=11.9545(9)       b=12.1069(6)      c=16.5916(7)
                alpha=80.316(4)    beta=71.254(5)    gamma=74.625(5)
Temperature:    100 K

                Calculated                  Reported
Volume          2183.5(2)                   2183.5(2)
Space group     P -1                        P -1
Hall group      -P 1                        -P 1
Moiety formula  C52 H34 Cu2 O11             C104 H68 Cu4 O22
Sum formula     C52 H34 Cu2 O11             C104 H68 Cu4 O22
Mr              961.89                      1923.85
Dx,g cm-3       1.463                       1.463
Z               2                           1
Mu (mm-1)       0.000                       0.000
F000            0.0                         362.8
F000'           983.84
h,k,lmax        14,15,20                    14,15,20
Nref            8956                        8267
Tmin,Tmax
Tmin'

Correction method= Not given

Data completeness= 0.923          Theta(max)= 0.900

                                                   wR2(reflections)=
R(reflections)= 0.1687( 6182)                      0.3478( 8267)

S = 1.037                 Npar= 595
```

The following ALERTS were generated. Each ALERT has the format
**test-name_ALERT_alert-type_alert-level.**
Click on the hyperlinks for more details of the test.

## Alert level A

PLAT029_ALERT_3_A _diffrn_measured_fraction_theta_full value Low . 0.936 Why?

**Author Response: The data are from single-axis rotation electron diffraction on a sample crystallising in P-1. Combined with blocky crystals having preferred orientations, 100% completeness has not been achieved within the constraints placed by the quality of the data available for merging.**

## Alert level B

RINTA01_ALERT_3_B The value of Rint is greater than 0.18
Rint given 0.201

**Author Response: The data constitue the merging of five electron diffraction data- sets and the Rint of the resulting dataset is entirely typical. This is owed to different impacts of dynamical effects across the various samples serving to significantly reduce internal agreement. These are expected to average out in merging to improve ultimate model statistics.**

PLAT020_ALERT_3_B The Value of Rint is Greater Than 0.12 ......... 0.201 Report

**Author Response: The data constitue the merging of five electron diffraction data- sets and the Rint of the resulting dataset is entirely typical, if not good. This is owed to different impacts of dynamical effects across the various samples serving to significantly reduce internal agreement. These are expected to average out in merging to improve ultimate model statistics.**

PLAT082_ALERT_2_B High R1 Value .................................. 0.17 Report

**Author Response: The data constitue the merging of five electron diffraction data- sets and the R1 of the resulting fit is entirely typical, if not good. This is owed to different impacts of dynamical effects across the various samples serving to significantly reduce internal agreement. Care has been taken to reject reflections that are more significantly affected by these effect and ensure that the model is robust in this context.**

PLAT420_ALERT_2_B D-H Bond Without Acceptor O1 --H1A . Please Check

**Author Response: This does not appear to be in error. There is nothing [else] in the structure that would appear to offer an H-bond acceptor, and no evidence of (nor room for) an included solvate, while charge balancing would require this be part of a neutral water molecule, and not an erroneously assigned hydroxyl. Any other position for this proton would generate physically unrealistic close contacts.**

```
PLAT911_ALERT_3_B Missing FCF Refl Between Thmin & STh/L=    0.600        508 Report
                2  0  0,   5  0  0,   7  0  0,   8  0  0,   0  1  0,   1  1  0,
                7  1  0,   8  1  0,   9  1  0,   0  2  0, -12  3  0,   0  3  0,
              -13 -6  1, -13  0  1,  -4  0  1,   1  0  1,   0  1  1,   3  1  1,
               11  3  1, -10 -5  2, -13 -4  2, -13 -3  2, -10 -3  2, -13 -2  2,
                5 -2  2,   7 -1  2, -11  0  2,  -7  0  2,  -4  0  2,   0  0  2,
                  (  478 More Missing: see the .ckf listing file)
```

**Author Response: The data are from single-axis rotation electron diffraction on a sample crystallising in P-1. Combined with blocky crystals having preferred orientations, 100% completeness has not been achieved within the constraints placed by the quality of the data available for merging. In addition, 63 reflections have been rejected in the final stages of the refinement, being either erroneously null measured or appearing more significantly affected by multiple diffraction from inspection of Fo-Fc plots and their impact on the refinement.**

```
PLAT922_ALERT_1_B wR2 in the CIF and FCF Differ by ..............     0.0072 Check
```

**Author Response: The origin of this difference comes from how and when EXTI is applied between the various reporting and validating stages of Olex2 and CheckCIF / Platon. In refinement, Fc is modified but, to make validation easier, EXTI is transfered from Fc^2 to Fo^2 which can lead to these discrepancies.**

---

**Alert level C**

```
PLAT041_ALERT_1_C Calc. and Reported SumFormula    Strings  Differ     Please Check
              Calc: C52 H34 Cu2 O11
              Rep.: C104 H68 Cu4 O22
PLAT042_ALERT_1_C Calc. and Reported MoietyFormula Strings  Differ     Please Check
              Calc: C52 H34 Cu2 O11
              Rep.: C104 H68 Cu4 O22
PLAT084_ALERT_3_C High wR2 Value (i.e. > 0.25) ..................        0.35 Report
PLAT220_ALERT_2_C NonSolvent   Resd 1  C   Ueq(max)/Ueq(min) Range        4.9 Ratio
PLAT220_ALERT_2_C NonSolvent   Resd 1  O   Ueq(max)/Ueq(min) Range        3.4 Ratio
PLAT250_ALERT_2_C Large U3/U1 Ratio for <U(i,j)> Tensor(Resd    1)        2.5 Note
PLAT341_ALERT_3_C Low Bond Precision on  C-C Bonds ...............    0.01165 Ang.
PLAT411_ALERT_2_C Short Inter H...H Contact  H4B      ..H25A     .       2.10 Ang.
                                                    x,1+y,z  =      1_565 Check
PLAT411_ALERT_2_C Short Inter H...H Contact  H16A     ..H22A     .       2.11 Ang.
                                                  -x,-y,1-z  =      2_556 Check
PLAT413_ALERT_2_C Short Inter XH3 .. XHn     H1BA     ..H1AA     .       2.06 Ang.
                                                    x,2+y,z  =      1_575 Check
```

```
PLAT413_ALERT_2_C Short Inter XH3 .. XHn     H1AC     ..H13A      .       2.09 Ang.
                                             -x,-1-y,2-z  =      2_547 Check
PLAT906_ALERT_3_C Large K Value in the Analysis of Variance ......     60.343 Check
PLAT906_ALERT_3_C Large K Value in the Analysis of Variance ......      8.754 Check
PLAT906_ALERT_3_C Large K Value in the Analysis of Variance ......      3.742 Check
PLAT906_ALERT_3_C Large K Value in the Analysis of Variance ......      2.401 Check
PLAT921_ALERT_1_C R1 in CIF and R1 Calcd from FCF Data Differ by .     0.0029 Check
PLAT925_ALERT_1_C The Reported and Calculated Rho(max) Differ by .       1.01 eA-3
```

---

### Alert level G

```
ABSMU01_ALERT_1_G  Calculation of _exptl_absorpt_correction_mu
               not performed for this radiation type.
PLAT002_ALERT_2_G Number of Distance or Angle Restraints on AtSite         65 Note
PLAT003_ALERT_2_G Number of Uiso or U(i,j)  Restrained non-H-Atoms         65 Report
PLAT004_ALERT_5_G Polymeric Structure Found with Maximum Dimension          1 Info
PLAT045_ALERT_1_G Calculated and Reported Z Differ by a Factor ...          2 Check
PLAT172_ALERT_4_G The CIF-Embedded .res File Contains DFIX Records          1 Report
PLAT176_ALERT_4_G The CIF-Embedded .res File Contains SADI Records         17 Report
PLAT187_ALERT_4_G The CIF-Embedded .res File Contains RIGU Records          1 Report
PLAT190_ALERT_3_G A Non-default RIGU Restraint Value for First Par     0.0020 Report
PLAT190_ALERT_3_G A Non-default RIGU Restraint Value for SecondPar     0.0020 Report
PLAT371_ALERT_2_G Long   C(sp2)-C(sp1) Bond  C5B      - C18B      .       1.41 Ang.
PLAT371_ALERT_2_G Long   C(sp2)-C(sp1) Bond  C7A      - C9A       .       1.41 Ang.
PLAT371_ALERT_2_G Long   C(sp2)-C(sp1) Bond  C10A     - C11A      .       1.42 Ang.
PLAT432_ALERT_2_G Short Inter X...Y Contact  O5B      ..C14B      .       3.01 Ang.
                                                x,y,-1+z  =      1_554 Check
PLAT720_ALERT_4_G Number of Unusual/Non-Standard Labels ..........         10 Note
              H2BA    H2BB    H1BA    H1BB    H1BC    H2AA    H2AB    H1AA
              H1AB    H1AC
PLAT768_ALERT_4_G RES Embedded Explicitly Supplied Scattering Data          4 Note
PLAT769_ALERT_4_G CIF Embedded Explicitly Supplied Scattering Data          4 Note
PLAT802_ALERT_4_G CIF Input Record(s) with more than 80 Characters          1 Info
PLAT860_ALERT_3_G Number of Least-Squares Restraints .............        588 Note
PLAT912_ALERT_4_G Missing # of FCF Reflections Above STh/L=  0.600        218 Note
PLAT933_ALERT_2_G Number of HKL-OMIT Records in Embedded .res File         63 Note
              -11  0  2, -11  3  6, -10 -5  2, -10 -3  2,  -7 -4  3,  -7  0  2,
               -6 -3  3,  -6  2  2,  -4  0  1,  -4  0  2,  -4  0  4,  -2 10  4,
                0 -3  3,   0  0  2,   0  0  6,   0  1  0,   0  1  1,   0  2  0,
                0  3  0,   0  3  3,   0  5  5,   0  6  6,   1  1  0,   2  0  0,
                2  4 10,   3 -6  3,   3 -3  9,   3  1  1,   5 -2  2,   5  0  0,
                5  1  2,   6  2  4,   6  4  4,   7 -1  2,   7  0  0,   7  1  0,
                7  1  2,   7  4 19,   8  0  0,   8  1  0,   8  1 15,   8  2  2,
                8  2  4,   8  3 13,   9 -5  6,   9  1  0,  10 -6  6,  10  4  7,
               11 -6  7,  11  3  1,
PLAT948_ALERT_5_G Externally Supplied Scattering Factors       CIF          4 Note
PLAT948_ALERT_5_G Externally Supplied Scattering Factors       RES          4 Note
PLAT969_ALERT_5_G The 'Henn et al.' R-Factor-gap value ..........      2.510 Note
              Predicted wR2: Based on SigI**2 13.57 or SHELX Weight 32.65
PLAT978_ALERT_2_G Number C-C Bonds with Positive Residual Density.          0 Info
```

---

1 **ALERT level A** = Most likely a serious problem - resolve or explain
6 **ALERT level B** = A potentially serious problem, consider carefully
17 **ALERT level C** = Check. Ensure it is not caused by an omission or oversight
25 **ALERT level G** = General information/check it is not something unexpected

```
 7 ALERT type 1 CIF construction/syntax error, inconsistent or missing data
17 ALERT type 2 Indicator that the structure model may be wrong or deficient
13 ALERT type 3 Indicator that the structure quality may be low
 8 ALERT type 4 Improvement, methodology, query or suggestion
 4 ALERT type 5 Informative message, check
```

---

---

It is advisable to attempt to resolve as many as possible of the alerts in all categories. Often the minor alerts point to easily fixed oversights, errors and omissions in your CIF or refinement strategy, so attention to these fine details can be worthwhile. In order to resolve some of the more serious problems it may be necessary to carry out additional measurements or structure refinements. However, the purpose of your study may justify the reported deviations and the more serious of these should normally be commented upon in the discussion or experimental section of a paper or in the "special_details" fields of the CIF. checkCIF was carefully designed to identify outliers and unusual parameters, but every test has its limitations and alerts that are not important in a particular case may appear. Conversely, the absence of alerts does not guarantee there are no aspects of the results needing attention. It is up to the individual to critically assess their own results and, if necessary, seek expert advice.

**Publication of your CIF in IUCr journals**

A basic structural check has been run on your CIF. These basic checks will be run on all CIFs submitted for publication in IUCr journals (*Acta Crystallographica*, *Journal of Applied Crystallography*, *Journal of Synchrotron Radiation*); however, if you intend to submit to *Acta Crystallographica Section C* or *E* or *IUCrData*, you should make sure that full publication checks are run on the final version of your CIF prior to submission.

**Publication of your CIF in other journals**

Please refer to the *Notes for Authors* of the relevant journal for any special instructions relating to CIF submission.

---

**PLATON version of 04/06/2025; check.def file version of 30/05/2025**

**Datablock CuEtOMOP_12Hz_pMOPH2O** - ellipsoid plot

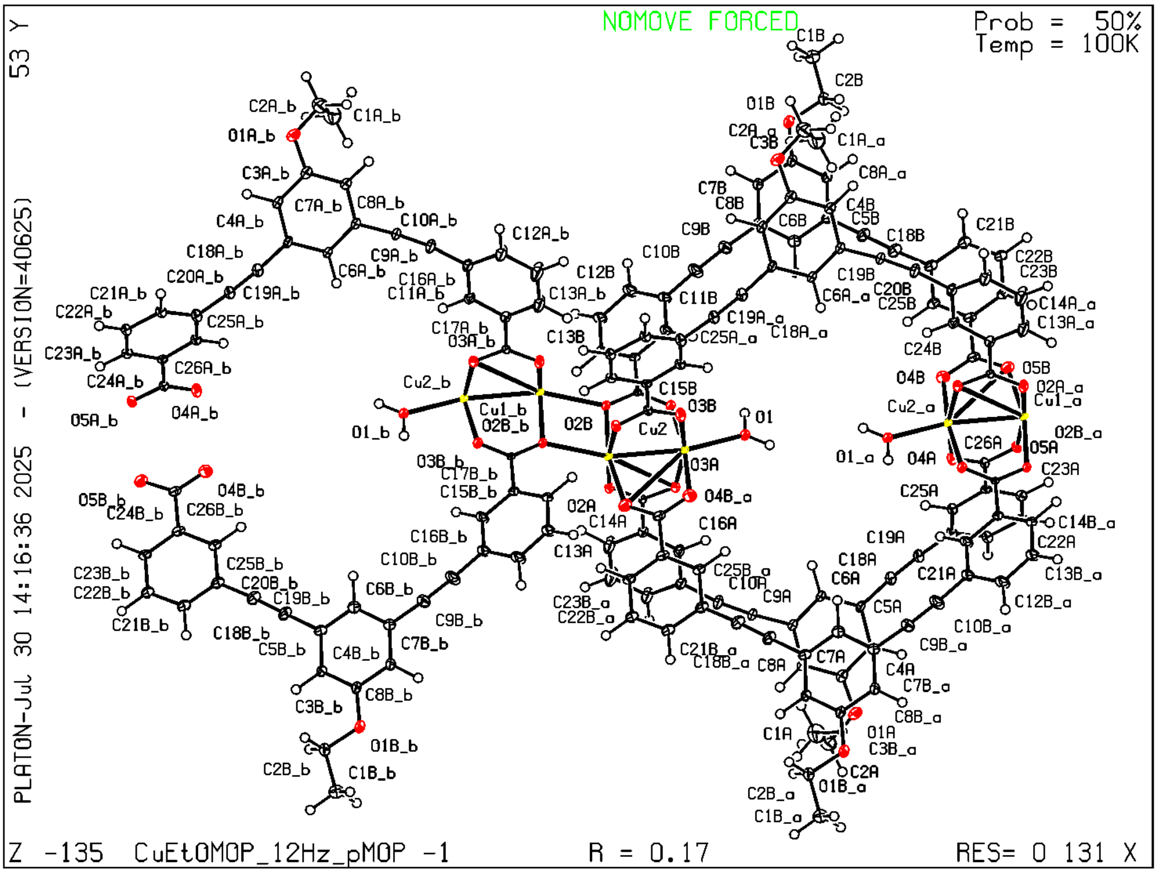

Supplement: Supplementary file 2 — Supporting information [file CuEtOMOP_12Hz_pMOPH2O_checkcif.pdf › Crystallographic_files_for_upload/CuEtOMOP_12Hz_pMOPH2O_checkcif.pdf]
