# Supplementary material for: A Metal‐Organic Polyhedron‐to‐Coordination Polymer Transition Revealed by 3D Electron Diffraction
Source: Angew Chem Int Ed Engl. 2025 Sep 6;64(43):e202514527. doi: 10.1002/anie.202514527 (PMC12535363; doi:10.1002/anie.202514527)

checkCIF/PLATON report

Structure factors have been supplied for datablock(s) CuEtOMOP_pMOP_activated_ordered

THIS REPORT IS FOR GUIDANCE ONLY. IF USED AS PART OF A REVIEW PROCEDURE FOR PUBLICATION, IT SHOULD NOT REPLACE THE EXPERTISE OF AN EXPERIENCED CRYSTALLOGRAPHIC REFEREE.

No syntax errors found.   CIF dictionary   Interpreting this report

Datablock: CuEtOMOP_pMOP_activated_ordered

Bond precision: C-C = 0.0142 A   Wavelength=0.02510

Cell:	a=14.9348(7)	b=16.2954(9)	c=19.0622(8)
	alpha=76.688(4)	beta=86.248(4)	gamma=73.103(4)
Temperature:	150 K		

	Calculated	Reported
Volume	4319.6(4)	4319.6(4)
Space group	P -1	P -1
Hall group	-P 1	-P 1
Moiety formula	C104 H64 Cu4 O20	C104 H64 Cu4 O20
Sum formula	C104 H64 Cu4 O20	C104 H64 Cu4 O20
Mr	1887.76	1887.82
Dx,g cm-3	1.451	1.451
Z	2	2
Mu (mm-1)	0.000	0.000
F000	0.0	713.4
F000'	1927.69	
h,k,lmax	17,19,22	17,19,22
Nref	15465	14617
Tmin,Tmax		0.250,1.000
Tmin'		

Correction method= # Reported T Limits: Tmin=0.250 Tmax=1.000
AbsCorr = MULTI-SCAN

Data completeness= 0.945   Theta(max)= 0.860

R(reflections)= 0.1757(8656)   wR2(reflections)= 0.4260(14617)

S = 1.066   Npar= 1180

The following ALERTS were generated. Each ALERT has the format
test-name_ALERT_alert-type_alert-level.
Click on the hyperlinks for more details of the test.

Alert level B

RINTA01_ALERT_3_B The value of Rint is greater than 0.18
Rint given 0.236

Author Response: The data constitue the merging of five electron diffraction data- sets and the Rint of the resulting dataset is entirely typical. This is owed to different impacts of dynamical effects across the various samples serving to significantly reduce internal agreement. These are expected to average out in merging to improve ultimate model statistics. This is in addition to being a crystal that has been treated to undergo a phase transformation via dehydration and fairly significant movement of the chains with respect to one another.

PLAT020_ALERT_3_B The Value of Rint is Greater Than 0.12 .... 0.236 Report

Author Response: The data constitue the merging of five electron diffraction data- sets and the Rint of the resulting dataset is entirely typical. This is owed to different impacts of dynamical effects across the various samples serving to significantly reduce internal agreement. These are expected to average out in merging to improve ultimate model statistics. This is in addition to being a crystal that has been treated to undergo a phase transformation via dehydration and fairly significant movement of the chains with respect to one another.

PLAT029_ALERT_3_B _diffrn_measured_fraction_theta_full value Low . 0.950 Why?

Author Response: The data are from single-axis rotation electron diffraction on a sample crystallising in P-1. Combined with blocky crystals having preferred orientations, 100% completeness has not been achieved within the constraints placed by the quality of the data available for merging.

PLAT082_ALERT_2_B High R1 Value .... 0.18 Report

Author Response: The data constitue the merging of five electron diffraction data- sets and the R1 of the resulting fit is entirely typical, if not good. This is owed to different impacts of dynamical effects across the various samples serving to significantly reduce internal agreement. Care has been taken to reject reflections that are more significantly affected by these effect and ensure that the model is robust in this context. This is in addition to being a crystal that has been treated to undergo a phase transformation via dehydration and fairly significant movement of the chains with respect to one another.

```
PLAT084_ALERT_3_B High wR2 Value (i.e. > 0.25) ..................       0.43 Report
```

Author Response: The data constitue the merging of five electron diffraction data- sets and the wR2 of the resulting fit is entirely typical. This is owed to different impacts of dynamical effects across the various samples serving to significantly reduce internal agreement. Care has been taken to reject reflections that are more significantly affected by these effect and ensure that the model is robust in this context. This is in addition to being a crystal that has been treated to undergo a phase transformation via dehydration and fairly significant movement of the chains with respect to one another.

```
PLAT220_ALERT_2_B NonSolvent   Resd 1   C   Ueq(max)/Ueq(min) Range        6.4 Ratio
```

Author Response: This corresponds to a highly labile ethoxy substituent off the centre of a conjugated ligand system. Disorder of the component has already been modelled to a reasonable extent.

Alert level C

```
PLAT220_ALERT_2_C NonSolvent   Resd 1   O   Ueq(max)/Ueq(min) Range        3.7 Ratio
```

Author Response: This corresponds to a highly labile ethoxy substituent off the centre of a conjugated ligand system. Disorder of the component has already been modelled to a reasonable extent.

```
PLAT222_ALERT_3_C NonSolvent Resd 1  H   Uiso(max)/Uiso(min) Range        6.5 Ratio
PLAT241_ALERT_2_C High   'MainMol' Ueq as Compared to Neighbors of        O3B Check
PLAT250_ALERT_2_C Large U3/U1 Ratio for <U(i,j)> Tensor(Resd    1)        2.1 Note
PLAT341_ALERT_3_C Low Bond Precision on  C-C Bonds ..............    0.01415 Ang.
PLAT411_ALERT_2_C Short Inter H...H Contact  H8D      ..H25A     .       2.00 Ang.
                                                   x,-1+y,1+z  =      1_546 Check
PLAT413_ALERT_2_C Short Inter XH3 .. XHn     H1BC     ..H1AB     .       2.12 Ang.
                                                   -1+x,y,1+z  =      1_456 Check
PLAT601_ALERT_2_C Unit Cell Contains Solvent Accessible VOIDS   <=         32 Ang**3
PLAT721_ALERT_1_C Bond    Calc     1.09(8), Rep     1.07700 Dev...       0.01 Ang.
              C1CB     -H1CE           1_555   1_555 ........     #  224 Check
PLAT722_ALERT_1_C Angle   Calc     111(10), Rep      109.50 Dev...       1.50 Degree
              H1CF    -C1CB    -H1CD       1_555  1_555  1_555     #  413 Check
PLAT722_ALERT_1_C Angle   Calc     108(11), Rep      109.50 Dev...       1.50 Degree
              H1CF    -C1CB    -H1CE       1_555  1_555  1_555     #  414 Check
PLAT906_ALERT_3_C Large K Value in the Analysis of Variance ......     67.694 Check
PLAT906_ALERT_3_C Large K Value in the Analysis of Variance ......     10.484 Check
PLAT906_ALERT_3_C Large K Value in the Analysis of Variance ......      4.114 Check
PLAT906_ALERT_3_C Large K Value in the Analysis of Variance ......      2.508 Check
PLAT911_ALERT_3_C Missing FCF Refl Between Thmin & STh/L=    0.597        775 Report
               -1  1  0,  -1  2  0,   0  2  0,  -2  3  0,  -1  3  0,  -3  4  0,
               -2  4  0,  -4  5  0,  -3  5  0,  -2  5  0,  10  5  0,  -5  6  0,
               -4  6  0,  -3  6  0,  -2  6  0,  -5  7  0,  -4  7  0,  -3  7  0,
               -6  8  0,  -5  8  0,  -4  8  0,  -3  8  0,  -7  9  0,  -6  9  0,
```

```
            -5  9  0,  -4  9  0,  -8 10  0,  -7 10  0,  -6 10  0,  -5 10  0,
                (  745 More Missing: see the .ckf listing file)
```

Alert level G

```
ABSMU01_ALERT_1_G  Calculation of _exptl_absorpt_correction_mu
                not performed for this radiation type.
PLAT002_ALERT_2_G Number of Distance or Angle Restraints on AtSite        126 Note
PLAT003_ALERT_2_G Number of Uiso or U(i,j)  Restrained non-H-Atoms        130 Report
PLAT004_ALERT_5_G Polymeric Structure Found with Maximum Dimension          1 Info
PLAT072_ALERT_2_G SHELXL First  Parameter in WGHT  Unusually Large       0.14 Report
PLAT073_ALERT_1_G H-atoms ref., but hydrogen treatment Reported as     constr Check
PLAT154_ALERT_1_G The s.u.'s on the Cell Angles are Equal ..(Note)      0.004 Degree
PLAT172_ALERT_4_G The CIF-Embedded .res File Contains DFIX Records          1 Report
PLAT176_ALERT_4_G The CIF-Embedded .res File Contains SADI Records         16 Report
PLAT187_ALERT_4_G The CIF-Embedded .res File Contains RIGU Records          1 Report
PLAT301_ALERT_3_G Main Residue  Disorder ..............(Resd    1)         2% Note
PLAT343_ALERT_2_G Unusual sp?      Angle Range in Main Residue for       C19C Check
PLAT371_ALERT_2_G Long   C(sp2)-C(sp1) Bond  C10B     - C11B     .       1.42 Ang.
PLAT371_ALERT_2_G Long   C(sp2)-C(sp1) Bond  C10D     - C11D     .       1.41 Ang.
PLAT371_ALERT_2_G Long   C(sp2)-C(sp1) Bond  C19D     - C20D     .       1.41 Ang.
PLAT410_ALERT_2_G Short Intra H...H Contact  H4C      ..H2CC     .       1.84 Ang.
                                                      x,y,z  =      1_555 Check
PLAT410_ALERT_2_G Short Intra H...H Contact  H2DA     ..H8D      .       1.76 Ang.
                                                      x,y,z  =      1_555 Check
PLAT410_ALERT_2_G Short Intra H...H Contact  H8D      ..H2DC     .       1.80 Ang.
                                                      x,y,z  =      1_555 Check
PLAT720_ALERT_4_G Number of Unusual/Non-Standard Labels ..........         34 Note
              H1BA    H1BB    H1BC    H2BA    H2BB    H2DA    H2DB    H2DC
              H2DD    H1AA    H1AB    H1AC    H2AA    H2AB    C1DB    H1DA
              H1DB    H1DC    H2CA    H2CB    H2CC    H2CD    C1CA    H1CA
              H1CB    H1CC    C1CB    H1CD    H1CE    H1CF    C1DA    H1DD
              H1DE    H1DF
PLAT768_ALERT_4_G RES Embedded Explicitly Supplied Scattering Data          4 Note
PLAT769_ALERT_4_G CIF Embedded Explicitly Supplied Scattering Data          4 Note
PLAT779_ALERT_4_G Suspect or Irrelevant (Bond) Angle(s) in CIF ...      39.30 Deg.
              H2CC -C2C  -H2CB    1_555   1_555   1_555 ......     #  387 Check
PLAT779_ALERT_4_G Suspect or Irrelevant (Bond) Angle(s) in CIF ...      42.80 Deg.
              C1CA -C2C  -H2CD    1_555   1_555   1_555 ......     #  396 Check
PLAT804_ALERT_5_G Number of ARU-Code Packing Problem(s) in PLATON           1 Info
PLAT860_ALERT_3_G Number of Least-Squares Restraints ............        1184 Note
PLAT933_ALERT_2_G Number of HKL-OMIT Records in Embedded .res File         79 Note
              -13 -1  3,  -9  0  3,  -8 -4  6,  -7-15  6,  -7 -3  5,  -7 -2  1,
               -7  1  3,  -6 -6  6,  -6  0  2,  -5 -5  5,  -5 -1  1,  -5  2  3,
               -4 14  2,  -3 -4  7,  -3 11  1,  -2 -7 10,  -2 -4  6,  -2  0  2,
               -2  1  1,  -1 -4  5,  -1 -1  1,  -1  0  1,  -1  4  3,  -1  6  5,
                0 -8  4,   0 -6  6,   0 -6  8,   0 -4  2,   0 -4  8,   0 -2  4,
                0 -1  2,   0 -1  8,   0  1  2,   0  2  0,   0  3  8,   0  9  4,
                1 -1  0,   1  2  5,   2 -6  4,   2 -4  2,   2  1  3,   2  3  2,
                3 -8  5,   3 -6  3,   3  9  9,   4 -8  4,   4 -6 10,   4  2  8,
                4  2  9,   4  4  4,
PLAT948_ALERT_5_G Externally Supplied Scattering Factors       CIF          4 Note
PLAT948_ALERT_5_G Externally Supplied Scattering Factors       RES          4 Note
PLAT969_ALERT_5_G The 'Henn et al.' R-Factor-gap value ..........       2.716 Note
              Predicted wR2: Based on SigI**2 15.68 or SHELX Weight 39.97
PLAT978_ALERT_2_G Number C-C Bonds with Positive Residual Density.          0 Info
```

```
 0 ALERT level A = Most likely a serious problem - resolve or explain
 6 ALERT level B = A potentially serious problem, consider carefully
16 ALERT level C = Check. Ensure it is not caused by an omission or oversight
30 ALERT level G = General information/check it is not something unexpected

 6 ALERT type 1 CIF construction/syntax error, inconsistent or missing data
20 ALERT type 2 Indicator that the structure model may be wrong or deficient
13 ALERT type 3 Indicator that the structure quality may be low
 8 ALERT type 4 Improvement, methodology, query or suggestion
 5 ALERT type 5 Informative message, check
```

It is advisable to attempt to resolve as many as possible of the alerts in all categories. Often the minor alerts point to easily fixed oversights, errors and omissions in your CIF or refinement strategy, so attention to these fine details can be worthwhile. In order to resolve some of the more serious problems it may be necessary to carry out additional measurements or structure refinements. However, the purpose of your study may justify the reported deviations and the more serious of these should normally be commented upon in the discussion or experimental section of a paper or in the "special_details" fields of the CIF. checkCIF was carefully designed to identify outliers and unusual parameters, but every test has its limitations and alerts that are not important in a particular case may appear. Conversely, the absence of alerts does not guarantee there are no aspects of the results needing attention. It is up to the individual to critically assess their own results and, if necessary, seek expert advice.

Publication of your CIF in IUCr journals

A basic structural check has been run on your CIF. These basic checks will be run on all CIFs submitted for publication in IUCr journals (*Acta Crystallographica*, *Journal of Applied Crystallography*, *Journal of Synchrotron Radiation*); however, if you intend to submit to *Acta Crystallographica Section C* or *E* or *IUCrData*, you should make sure that full publication checks are run on the final version of your CIF prior to submission.

Publication of your CIF in other journals

Please refer to the *Notes for Authors* of the relevant journal for any special instructions relating to CIF submission.

PLATON version of 04/06/2025; check.def file version of 30/05/2025

Datablock CuEtOMOP_pMOP_activated_ordered - ellipsoid plot

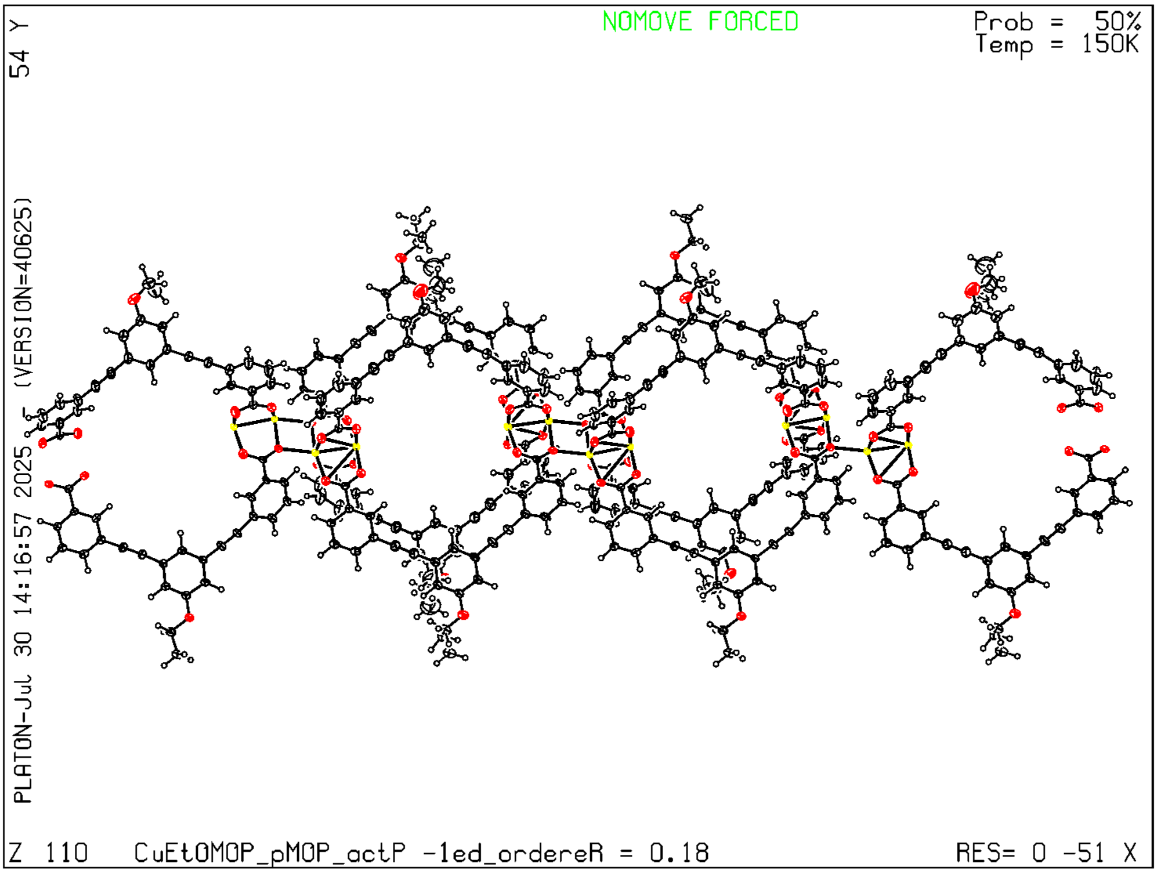

Supplement: Supplementary file 2 — Supporting information [file CuEtOMOP_pMOP_activated_ordered_checkcif.pdf › Crystallographic_files_for_upload/CuEtOMOP_pMOP_activated_ordered_checkcif.pdf]
